# Supplementary material for: Gastrointestinal and Intra-Abdominal Mucormycosis in Non-Haematological Patients—A Comprehensive Review
Source: J Fungi (Basel). 2025 Apr 9;11(4):298. doi: 10.3390/jof11040298 (PMC12028458; doi:10.3390/jof11040298)
Supplement: Supplementary file 1 [file jof-11-00298-s001.zip › jof-3534399-supplementary.pdf]

Gastrointestinal and intra-abdominal mucormycosis in non-haematological patients, a comprehensive review.

Supplementary files:

Table S1. Summary of epidemiological and clinical data reported in the 290 cases of gastrointestinal (GI) and intra-abdominal (IA) mucormycosis that were included in the study.

Table S2. Summary of diagnostic, treatment and outcome reported in the 290 cases of gastrointestinal (GI) and intra-abdominal (IA) mucormycosis that were included in the study.

Figure S1. Distribution (%) of the underling diseased and conditions according to the decade of publication.

Figure S2. Distribution (%) of main underlying diseases and conditions according to the country for the five countries with more than 10 reported cases.

	Author, Reference	Year	Country	Sex /	Age	Underlying condition	Symptoms	Endoscopy	Abd CT scan/USG	Infection site
1	Sutehreland et al. (30)<br>Rvw / (29)	1960	South Africa	M	50	Pulmonary tuberculosis	Abd pain	NR	NR	Stomach
2	Kahn et al. (31)	1963	South Africa	F	17	Diabetes, post-partum haemorrhage<br>Gastric tube	Abd distension, vomiting	NR	NR	Stomach
3	Abramowitz et al. (32)	1964	South Africa	M	37	Viscus perforation<br>Hydrocortisone	Abd pain, melena, vomiting with blood	NR	NR	Stomach
4	Dannheimer et al. (33)<br>Rvw /(29)	1974	Germany	M	46	Diabetes mellitus, liver cirrhosis	Abd pain	NR	NR	Stomach
5	Lawson et al. (34)	1974	South Africa	M	0,3	Kwashiorkor, viscus perforation	NR	NR	NR	Stomach
6	Lawson et al.	1974	South Africa	M	30	Peptic ulcer perforation	Dyspepsia	NR	NR	Stomach
7	Lawson et al.	1974	South Africa	M	35	Viscus perforation	Abdominal pain	NR	NR	Stomach
8	Lawson et al.	1974	South Africa	M	16	Typhoid fever	NR, typhoid fever	NR	NR	Stomach
9	Lawson et al.	1974	South Africa	M	41	Dyspepsia, history of hematemesis	Peritonitis or perforation	NR	NR	Stomach
10	Lawson et al.	1974	South Africa	M	33	Dyspepsia, history of hematemesis	Peritonitis or perforation	NR	NR	Stomach
11	Schulman et al. (35)<br>Rvw /(29)	1979	South Africa	M	23	NR	Abd pain, vomiting	NR	NR	Stomach
12	Brullet et al. (36)	1993	Spain	M	42	AIDS, CD4+ 50 cells/mm3<br>ICU, steroids	Abd pain	Gastric ulcer (body), oedematous margins, friable +granulomatous base	NR	Stomach
13	Winkler et al. (37)<br>Rvw /(29, 38)	1996	Austria	F	37	KT, cyclosporine A, steroids<br>Acute rejection / steroid pulse, anti-thymocyte globulin<br>IV iron, transfusions	Melena	Gastric ulcer, 3 cm, greyish exudate	NR	Stomach
14	Corley et al. (39)<br>Rvw / (29, 38)	1997	USA	M	53	H transplant	Anorexia, nausea, hiccups	Gastric green, necrotic ulcer (juncture of the antrum and body)	NR	Stomach
15	Knoop et al. (40)<br>Rvw / (29)	1998	Belgium	M	32	HLT/ cyclosporine, azathioprine MPD	Abd pain, hematemesis	Gastric (cardia) necrosis	NR	Stomach
16	Sharma et al. (41)	1998	India	M	52	NR	NR	NR	NR	Stomach
17	Sheu et al. (42)	1998	Taiwan	M	70	KT (1 M)	Abd pain, fever	Gastric (great curvature) ulcer	NR	Stomach

18	Barosso et al. (43) Rvw / (38)	1999	Spain	F	50	Diabetes mellitus	Abd pain, vomiting	Gastric necrotic ulcer (whole gastric circumference, fornix-body junction to prepyloric area), green exudate	NR	Stomach
19	Cherney et al. (44) Rvw / (29), (38)	1999	USA	M	69	Systemic vasculitis, glucocorticoids, cyclophosphamide Duodenal ulcer, Abd surgery	Anorexia, Abd pain	Green exudate with areas of necrosis	Air in the wall of the stomach.	Stomach
20	Al-Rikabi et al. (45) Rvw / (29)	2000	Saudi Arabia	M	55	Diabetes mellitus *H. pylori* chronic active gastritis	Abd pain, decreased appetite, weight loss	Gastric (fundus) ulcer	Thickening of the stomach wall	Stomach
21	Pickeral et al. (46) Rvw / (29)	2000	USA	M	66	COPD, chronic renal failure atherosclerosis (coronary, carotids) abdominal aortic aneurysm +placement of an artificial graft	Abd pain, nausea, vomiting	Gastric (cardia) 3 x 5 cm ulcer	NR	Stomach
22	Geramizadeh et al. (47)	2001	Iran	F	63	SLE, prednisone	Occult blood in stool	NR	NR	Stomach
23	Tinmouth et al. (48) Rvw / (29, 38)	2001	USA	M	58	KT, tacrolimus, prednisolone, MMF Steroid-induced diabetes	Abd pain, distention, constipation	Gastric (body) ulcer, necrosis, duodenal ulcers, necrosis	splenic abscess	Stomach
24	Paulo de Oliveira et Milech. (49) Rvw / (29)	2002	Brazil	F	17	Diabetes mellitus, ketoacidosis	Abd pain	Gastric (greater curvature, posterior wall) 10 cm ulceration, exudate	NR	Stomach
25	Park et al. (50) Rvw / (29, 38),	2002	South Korea	M	56	Chronic alcohol use Chronic HBV	Abd pain, vomiting	Gastric (fundus to mid body) ulcer, necrosis, bleeding	Gastric wall thickening, perigastric fat infiltration, regional lymph node enlargement	Stomach
26	Shahapure et al. (51) Rvw / (29, 38),	2002	India	M	35	Alcohol use	Abd pain, Acute abdomen	Gastric plaque ulceration	NR	Stomach
27	Vera et al. (52) Rvw / (29)	2002	England	M	45	LT (alcohol-induced cirrhosis) tacrolimus, hydrocortisone, azathioprine	NR	Gastric bleeding lesion	NR	Stomach
28	Maravi-Poma et al. [a] (53) Rvw / (29)	2004	Spain	F	50	ICU, pneumonia, ARDS, MOF, Corticosteroids	Massive upper gastrointestinal bleeding	Gastric ulcer, necrosis, green exudate, bleeding	NR	Stomach
29	Maravi-Poma et al. [a]	2004	Spain	M	45	ICU, Pneumonia, ARDS, MOF Alcoholism, malnutrition corticosteroids	Massive upper gastrointestinal bleeding	Gastric ulcer, necrosis, green exudate, bleeding	NR	Stomach
30	Maravi-Poma et al. [a]	2004	Spain	F	47	ICU, polytrauma, shock, sepsis, corticosteroids	NR	Gastric ulcer, necrosis, green exudate, bleeding	NR	Stomach

31	Maravi-Poma et al. [a]	2004	Spain	F	42	ICU, Pneumonia, ARDS, MOF, corticosteroids<br>Coagulopathy, schizophrenia	Massive upper Gastrointestinal bleeding	Gastric ulcer, necrosis, green exudate, bleeding	NR	Stomach
32	Maravi-Poma et al. [a]	2004	Spain	F	66	ICU, Pneumonia, ARDS, MOF, cardiopathy, corticosteroids	NR	NR	NR	Stomach
33	Stamm et al. (54)	2005	Switzerlan d	M	52	Fall trauma on telluric ground multiple fractures, Abd bleeding (retroperitoneum)<br>*Pseudomonas* sepsis, respiratory distress, renal failure	Gastric bleeding	NR	NR	Stomach
34	Prasad N. et al. (55)<br>Rvw /(29, 38),	2006	India	M	42	KT/prednisolone, ciclosporin, azathioprine<br>Alcohol use	Abd pain, hematemesis, melena	Gastric (body and antrum junction) ulceration, esophagitis	NR	Stomach
35	Devlin et al. (56)<br>Rvw / (29)	2007	USA	M	32	Abd surgery /Crohn Disease<br>Infliximab, 6-Mercaptopurine, Corticosteroids	Abd pain	NR	Pneumoperitoneum	Stomach
36	Ho et al. (57)<br>Rvw / (29)	2007	Taiwan	F	58	Chronic alcoholism<br>Glucose intolerance<br>Colic perforation	Abd pain	Gastric (body), 5 cm mass, ulcers	NR	Stomach
37	Vaiphei et al. (58)	2007	India	F	33	Obesity<br>ICU, Typhoid fever	Fever	NR	NR	Stomach
38	Chung et al. (59)<br>Rvw / (29)	2008	Taiwan	M	58	HIV CD4+ 257 cells/mL<br>Pneumonia and respiratory failure	Abd pain, abdominal fullness	NR	No abnormalities	Stomach
39	Shiva Prasad et al. (60)	2008	India	M	28	Gastro-enteritis episode<br>ICU, MV, dialysis	Haematochezia then gastric bleeding	Colic ulcers then gastric (lesser curvature) ulcer + necrosis	NR	Stomach
40	Azhar et al. (61)	2009	USA	M	43	Diabetes mellitus	Abd pain, vomiting	Gastric (cardia) ulcer	Hemoperitoneum, collapsed gastric lumen	Stomach
41	Nandu et al. (62)	2009	India	F	79	Diabetes mellitus, nephropathy	Melena	Gastric tumour like lesion, necrosis	NR	Stomach
42	Small et al. (63)<br>Rvw / (29)	2010	USA	M	60	ICU, MV	Abd pain	Gastric (antrum, cardia to the lesser curve) 50% ulcer, green exudate	Gastric body wall thickening	Stomach
43	Feng et al. (64)<br>Rvw / (29)	2010	Taiwan	M	57	Chronic hepatitis B, CAPD with irregular steroid use,<br>Septic shock	Abd pain, fever	Gastric brown-greenish ulcer	Intramural gas in the greater curvature side of the gastric body	Stomach
44	Johnson et al. (65)<br>Rvw / (38)	2010	USA	M	60	Diabetes mellitus, peripheral vascular disease,<br>Schizophrenia<br>Traffic accident, ICU	Melena	Gastritis, bleeding (cardiac, fundic)<br>Gastric ulcers (antrum, prepyloric)	NR	Stomach

45	Paydar et al. (66)<br>Rvw / (29)	2010	Iran	F	36	Diabetes mellitus	Abd pain, hematemesis, melena	Gastric (greater curvature) 10×15 cm ulcer, bleeding, congestion	NR	Stomach
46	Pruthvi et al. (67)<br>Rvw / (29)	2010	India	M	76	Urinary tract infection with septicaemia	Occult gastrointestinal bleeding	Gastric (lesser curve) 3 x 4 cm ulcer	NR	Stomach
47	Shenoi et al. (68)<br>Rvw/ (29)	2010	USA	M	14	SLE, nephrotic syndrome,<br>pulse cyclophosphamide + MPD, prednisolone, tacrolimus<br>ICU, septic shock, MV<br>Steroid-induced diabetes	Abd pain	Gastric walls serpiginous, purplish, nodular submucosal lesions	No clear abnormalities	Stomach
48	Woo et al. (69)	2010	Hong Kong	M	69	KT (cadaveric), corticosteroid and cyclosporin	Epigastric discomfort, fever, GI bleeding	Gastritis	NR	Stomach
49	Chhaya et al. (70)<br>Rvw / (29)	2011	England	F	53	Ulcerative colitis, oral prednisolone<br>Urosepsis	Melena	Gastric body plaque-like ulcers	NR	Stomach
50	Chang et al. (71)<br>Rvw / (29)	2012	South Korea	F	46	LT	Abd pain, fever	Cardia to mid gastric body > 10cm yellowish lesion	Thinned wall in stomach greater curvature of cardia	Stomach
51	Dutta et al. (72)<br>Rvw / (29, 73),	2012	India	F	64	NR	Abd distension, anorexia	Gastric erosive ulcers (fundus, body)	NR	Stomach
52	Muthuswamy et al. (74)<br>Rvw / (29)	2012	USA	M	52	COPD, ICU, MV, sepsis<br>Cytopenia (NS)	Gastric bleeding	Gastric (lower curvature) ulcer, necrosis	NR	Stomach
53	Ryan et al. (75)<br>Rvw / (38)	2012	Ireland	M	47	Diabetes,<br>HIV, CD4+ 350 cells/mm3<br>Cirrhosis / HCV<br>ICU	Haematemesis, melena	Gastric (fundus) unmovable blood clot then (d 7) fundic grey-coloured ulcer	Gastric mucosal discontinuity	Stomach
54	Corey et al. (76)	2013	USA	F	55	Diabetes mellitus<br>Autoimmune hepatitis, prednisone<br>End-stage liver disease and cirrhosis / portal hypertension / trans jugular intrahepatic portosystemic shunt (TIPS)	Abd pain, melena	Gastric (fundus) 2 cm ulcer, necrosis	Cecum + ascending colon wall thickening, peri colonic fat stranding, surrounding sub-centimetric lymph nodes. Cirrhosis with splenomegaly, TIPS in the proper position	Stomach
55	Irtan et al. (77)<br>Rvw /(29)	2013	France	F	4	Multivisceral transplantation<br>half of the stomach, duodeno-pancreas, small bowel, and right colon<br>basiliximab, tacrolimus, MPD	Hematemesis	NR	NR before surgery<br>Normal for the follow up	Stomach
56	Katta et al. (78)<br>Rvw / (29)	2013	USA	M	60	HVC hepatitis<br>loss of consciousness on his farm<br>ICU, intubation	Abd pain	Gastric (cardia to fundus) 27 cm ulcer	Stomach wall thickening	Stomach

57	Machicado et al. (79)<br>Rvw / (29)	2013	USA	M	48	Traffic accident, Abd trauma<br>ICU, corticosteroids<br>Ileal perforation, surgery	Vomiting, melena	Gastric ulcers (body, antrum, fundus)	NR	Stomach
58	Bini et al. (80)<br>Rvw / (29, 38)	2014	Italy	F	26	Diabetes mellitus,<br>Cholecystectomy, sepsis, MOF	Abd pain, haematemesis, fever	Gastric necrosis	Full-thickness oedema of the stomach	Stomach
59	Kaiser et al. (81)<br>Rvw / (29, 38),	2014	Switzerlan d	F	63	Diabetes<br>LT (hepatocellular carcinoma - chronic HBV)<br>MMF, MPD	GI bleeding	Gastric (fundus) necrotic area	NR	Stomach
60	Lee et al. (82)<br>Rvw / (29)	2014	South Korea	M	55	Alcoholic liver cirrhosis	Abd pain	NR	Gastric ulcer perforation, pneumoperitoneum, hemoperitoneum	Stomach
61	Kulkarni et al. (83) Rvw /(29)	2015	India	M	50	Diabetes mellitus	Abd pain, distension, nausea, vomiting, anorexia, fever	NR	Pneumoperitoneum	Stomach
62	Nandwani et al. (84) Rvw / (27, 29)	2015	India	M	45	KT / tacrolimus, MMF, basiliximab<br>ICU, septic shock	Abd pain, melena	Gastric (greater curvature) ulcer, necrosis	Gastric perforation, pneumoperitoneum	Stomach
63	Nasta et al. (85)<br>Rvw / (29)	2015	India	M	50	Diabetes<br>Absent spleen	Abd pain	NR	perforation of fundus, extravasation into left pleural space, rent in left hemi-diaphragm	Stomach
64	Raviraj et al. (86)<br>Rvw / (38)	2015	India	F	17	Haemolytic uremic syndrome<br>ICU, haemodialysis, plasmapheresis	Abd pain, vomiting, hematemesis	Gastric (oesophagus junction) bleeding ulcer	Gastric ulcer leakage	Stomach
65	Lin et al. (87)	2016	Taiwan	M	56	Diabetes mellitus	Hematemesis, melena	Gastric disrupted wall layers+ thickening, regional lymph nodes	Gastric wall thickening, regional lymph nodes	Stomach
66	Mittal et al. (88)<br>Rvw / (29)	2016	India	M	85	Diabetes mellitus	Abd pain, distension, vomiting	Gastric (body) ulcer, necrosis	Gastrocolic fistula	Stomach
67	Ravi et al. (89)<br>Rvw / (29)	2016	India	F	65	Diabetes mellitus, COPD<br>Hydropneumothorax, pleurisy /<br>*S. viridians* + *C. glabrata* pancreatitis	NR	NR	Gastric perforated ulcers, perforated diaphragm, liver abscess	Stomach
68	Tathe et al. (90)<br>Rvw / (27, 29, 38),	2016	India	M	56	Liver cirrhosis	Abd pain, distension, hematemesis	Gastric (greater curvature) 6 x 6 cm ulcer, necrosis, congested surrounding mucosa	NR	Stomach
69	Chow et al. (91)<br>Rvw / (29, 38)	2017	USA	M	34	HIV immune reconstitution inflammatory syndrome, prednisone<br>Gun shot, Abd surgery	NR	Gastric (cardia + fundus) erosions, necrosis	Stomach and pleural space communication	Stomach

70	Chugh et al. (92)<br>Rvw / (29)	2017	USA	M	36	Alcohol and drug use<br>Vasopressive shock	Melena	Gastric 5 cm necrotic ulcer, exudate	NR	Stomach
71	Galvan-Fernandez et al. (93)<br>Rvw / (27)	2017	Spain	M	61	Diabetes mellitus<br>Chronic renal failure, metabolic acidosis	NR	NR	Pneumoperitoneum, gastric wall thickening + emphysema	Stomach
72	Grimaldi et al. (94)	2017	Belgium	F	30	Soft-tissue abdominal and pelvic damage, pulmonary contusion, burns / terrorist bombing<br>ICU, sepsis<br>Impaired immune response (low absolute lymphocyte count, and monocyte HLA-DR expression, increased expression of programmed death-1 (PD-1) on T-cells)	NR	NR	Gastric and splenic necrosis	Stomach
73	Lyo et al.<br>Rvw / (29)	2017	USA	M	36	Alcohol use<br>Pneumonia<br>ICU / respiratory failure, MV	Vomiting, melena	Gastric (40% the body) necrotic ulcer	NR	Stomach
74	Metussin et al. (95)<br>Rvw / (29)	2017	Brunei	M	42	Diabetes mellitus<br>ICU	NR	Gastric (great curve, body ulcer, necrosis)	NR	Stomach
75	Nasa et al. (96)<br>Rvw / (29)	2017	India	M	31	Drug use, cerebral oedema<br>ICU	Abd pain, distension, vomiting, melena	Oesophageal + gastric ulcerations	Gastric perforation, pneumoperitoneum	Stomach
76	Sanchez-Velasquez et al. (97)<br>Rvw / (29, 38),	2017	Spain	F	53	ICU, MV<br>dexamethasone	Hematemesis	Gastric ulcer (esophagogastric junction to the upper stomach), bleeding	NR	Stomach
77	Suhaildeen et al. (98)<br>Rvw / (29)	2017	India	F	52	Diabetes mellitus	Abd pain, vomiting, appetite loss	Gastric (esophagogastric junction, cardia, fundus) ulcero-proliferative lesion	Stomach wall thickening, perigastric nodes	Stomach
78	Abreu et al. (99)<br>Rvw / (29)	2018	Brasil	F	23	Previous tuberculosis	Abd pain + distension, vomiting, fever	Gastritis	Gastric distension + diffuse parietal thickening	Stomach
79	Alfano et al. (100)<br>Rvw / (27, 29)	2018	Italy	F	42	KT + LT<br>MPD, tacrolimus, MMF	Abd pain, melena	Gastric body necrotic ulcers	NR	Stomach
80	Chang et al. (71)	2018	Taiwan	F	70	Diabetes mellitus	Abdominal pain, distension, fever	Gastric (esophagogastric junction, cardia) infiltrative + necrotic ulceration	NR	Stomach
81	Kim et al. (101)<br>Rvw / (29)	2018	South Korea	M	55	Diabetes mellitus,<br>KT	Abd pain, distension, vomiting	Gastric (antrum to upper body) erosive mucosa, necrosis	gastric wall thickening	Stomach

82	Termos et al. (102)<br>Rvw / (29)	2018	Kowait	F	52	Diabetes mellitus, chronic kidney disease/ dialysis<br>Drug abuse<br>ICU/arm cellulitis, sepsis	Abd pain	NR	Oedematous gastric wall, perforation	Stomach
83	Adhikari et al. (103)<br>Rvw /(27)	2019	Nepal	F	57	Tobacco use	Hematemesis, melena	Gastric (lesser curvature)<br>10 × 6 mm ulcer	NR	Stomach
84	Gani et al. (104)<br>Rvw / (29)	2019	USA	M	79	Diabetes mellitus,<br>KT/MMF, Tacrolimus, Prednisone	Dysphagia, odynophagia	Gastric ulcer, greenish exudate	NR	Stomach
85	Guzman Rojas et al. (105)	2019	USA	M	46	Diabetes mellitus with gastroparesis<br>HIV well controlled infection	Abd pain, nausea	Gastric (fundus) ulcer	Gastric wall thickening, perforation	Stomach
86	Lankarani et al. (106)<br>Rvw / (29)	2019	USA	F	54	Pure motor hemiplegia	Abd pain, nausea, vomiting, melena, fever	Gastric (fundus, proximal body) necrosis and oedema, bleeding (antrum) petechiae	NR	Stomach
87	Malek et al. (107)<br>Rvw / (29)	2019	USA	F	54	Diabetes mellitus<br>Renal dysfunction	Abd pain, hematemesis	NR	Gastric perforation, abdominal gas +fluid collection	Stomach
88	Peng et al. (108)<br>Rvw / (27)	2019	China	F	47	KT/ MPD, tacrolimus, steroids, MMF haemodialysis	Chest pain, melena	Oesophageal + gastric extensive erosion, necrosis, ulcer	NR	Stomach
89	Sharaan et al. (109)<br>Rvw / (29)	2019	USA	M	28	Alcohol use<br>Rhabdomyolysis<br>ICU	Abd pain	NR	Pneumoperitoneum	Stomach
90	Uchida et al. (38)<br>Rvw /(29)	2019	Japan	F	82	Adult-onset Still disease<br>MPD 1g/d for 3 d, tocilizumab (8mg/kg)	Abd pain, melena	Pylorus ulcers	No abnormalities	Stomach
91	Monte Junior et al. (110)<br>Rvw / (27, 29)	2020	Brazil	M	86	COVID-19<br>ICU hydrocortisone	Melena, fever	Gastric ulcers, haemorrhagic base	NR	Stomach
92	Hameed et al. (111)<br>Rvw / (29)	2020	India	F	22	Metabolic acidosis<br>ICU	Abd pain, distension, fever	NR	NR	Stomach
93	Jaju et al. (112)	2020	India	M	48	Alcohol, tobacco use<br>Metabolic acidosis post *P. falciparum* malaria	Haematemesis and melaena	Gastric ulcerated mass, bleeding	Fundal mucosa thickening	Stomach
94	Jung et al. (113)<br>Rvw / (27, 29)	2020	South Korea	F	41	Traffic accident, thoracic surgery<br>ECMO	Melena	Gastric ulcers, bleeding (fundus, body)	NR	Stomach
95	Naqvi et al. (73)<br>Rvw / (27, 29)	2020	USA	F	55	Diabetes mellitus<br>Chronic kidney disease	Abd pain, nausea	Gastric (fundus) ulcers, exudate	Transmural thickening perigastric inflammation, reactive adenopathy,	Stomach

96	Rivas et al. (114)<br>Rvw / (29)	2020	Scotland	M	40	Metabolic encephalopathy<br>Liver cirrhosis<br>ICU	Nausea/vomiting, diarrhoea	Gastric (fundus) ulcer	Gastric contained perforation, bowel ischemia	Stomach
97	Sharma et al. (115)<br>Rvw / (29)	2020	India	F	55	Diabetic ketoacidosis	Abd pain, fever	NR	Emphysematous gastritis greater curvature perforation	Stomach
98	Sharma et al.	2020	India	F	66	Chronic renal failure, haemodialysis	Hematemesis	Gastric body ulcer	Emphysematous gastritis	Stomach
99	Ghuman et al. (116)	2021	India	M	45	Alcohol use, pesticide ingestion	NR	NR	Non-enhancement in the fundus of the stomach, focal areas of pneumatosis.	Stomach
100	Huang et al. (117)<br>Rvw / (27, 29)	2021	China	M	16	NR	Abd discomfort, hematemesis	Gastric perforation<br>Bleeding	NR	Stomach
101	Huang et al.	2021	China	F	33	Traffic accident with limb trauma, metabolic acidosis, ICU, septic shock, MOF	NR	Gastric bleeding	NR	Stomach
102	Rai et al. (118)<br>Rvw / (27)	2021	India	F	36	Viscus perforation, peritonitis<br>Metabolic acidosis	Abd pain, distension, vomiting	NR	NR	Stomach
103	Yuvaraj et al. (119)	2021	India	M	53	Diabetes mellitus, alcohol abuse<br>COVID-19 pneumonia, steroids	Abd pain, hematemesis, melena	Gastric proliferative lesion (fundus) + blood and exudative material	Stomach exophytic ulcerative lesion; perigastric, perisplenic fat stranding; splenic infarction; then gastrosplenic fistula	Stomach
104	Bhaskar et al. (120)<br>Rvw / (29)	2022	India	F	22	Necrotic pancreatitis<br>ICU MOF<br>enteral feeding	Abd pain, fever	NR	Defect of posterior wall of stomach, communication with necrotic collection and sigmoid colon perforation	Stomach
105	Chauhan et al. (121)	2022	India	M	35	Down's syndrome, COVID-19 ICU, pulmonary embolism	NR	Gastric (body) necrotic ulcers	Gastric ulcers	Stomach
106	Danion et al. [b] (10)<br>Rvw / (122)	2022	France	M	66	COVID-19, ICU, MV, corticosteroids	Abd pain, distension, gastric bleeding	Gastric infiltration + ulcer	Not conclusive	Stomach
107	Khsiba et al. (123)<br>Rvw / (29)	2022	Tunisia	F	61	Diabetes mellitus, dyslipidaemia, family history of peptic ulcer<br>4 M history of dry cough, inhaled corticosteroids	Vomiting	Gastric ulcer (fundus) + budding greyish infiltration	Fundic mucosal thickening	Stomach
108	Khsiba et al.	2022	Tunisia	M	59	NR	Abd pain, vomiting, fever, ascites	Gastric ulcer, fragile mucosa, bleeding	USG: unremarkable except ascites	Stomach
109	Sachan et al. (124)<br>Rvw/ (29)	2022	India	M	58	NR	Dysphagia and recurrent melena	Gastric body ulceration + black coloured polypoid growth	NR	Stomach

110	Sachan et al.	2022	India	F	18	Diabetes, ketoacidosis<br>ICU, MV	Anaemia	Gastric black coloured mucosa	NR	Stomach
111	Safwan et al. (125)<br>Rvw / (29)	2022	India	M	45	Diabetes mellitus<br>Chronic liver disease (Child-Pugh C), ICU	Vomiting, melena	Gastric (fundus, body) ulceration	NR	Stomach
112	Albtoosh et al. (126)<br>Rvw / (29)	2023	Jordan	M	46	NR, tobacco use	Abd pain, hematemesis, melena	Gastric body ulcer below the gastroesophageal junction	No abnormalities	Stomach
113	Arora et al. (127)	2023	India	M	53	COVID-19 (DG 1,5 M ago)<br>ICU, SDRA, hydrocortisone<br>Pneumothorax	GI bleeding	Gastric ulcer (fundus)	Non-enhancing stomach fundus wall adherent to the left hemidiaphragm, gastro pleural connection	Stomach
114	Bhowmik et al. (128)<br>Rvw / (29)	2023	India	M	48	Traffic accident, septicaemia, enteral feeding	NR	Pylorus necrotic ulcer, exudates	NR	Stomach
115	Khanna et al. (129)<br>Rvw / (29)	2023	India	M	43	COVID-19 (DG 1 M ago)	Hematemesis, melena	Gastric body necrotic ulcer	NR	Stomach
116	Kim et al. (130)<br>Rvw / (29)	2023	USA	M	57	Diabetes mellitus<br>KT + PT	Abd pain, vomiting, fever	Gastric (antrum, cardia, body) necrosis	gastric pneumatosis	Stomach
117	Malakar et al. (131)<br>Rvw / (29)	2023	India	M	82	COVID-19 (DG 2 M ago), prednisolone for 21 d	Hematemesis, melena	Gastric 8 cm ulcer	NR	Stomach
118	Marco et al. (132)	2023	Spain	M	53	ICU (loss of consciousness)<br>MOF, metabolic acidosis<br>hydrocortisone	Gastric bleeding	Gastric greater curvature + fibro-necrotic material	Gastric wall necrosis	Stomach
119	Chaudhari et al. (133)<br>Rvw / (29)	2024	India	M	50	Sepsis with MOF	Hematemesis	Gastric (fundus) 3 cm proliferative lesion, greenish exudate	No gastric abnormalities	Stomach
120	de Lucena et al. (134)	2024	Brazil	F	29	Diabetes mellitus, ketoacidosis	NR	Gastric (fornix) ulcer and infiltration	No abnormalities	Stomach
121	He et al. (135)	2024	China	M	57	ANCA associated vasculitis, haemodialysis<br>COVID19, prednisone	Melena	Gastric ulcers	NR	Stomach
122	Huang et al. (136)	2024	China	F	63	NR	Abd pain, distension	Gastric mucosal swelling, necrosis	Gastric body great curvature wall thickening	Stomach
123	Ji R. (137)	2024	China	M	57	Liver cirrhosis<br>Traffic accident, Abd surgery (splenectomy, cholecystectomy and choledocholithotomy<br>ICU	Bloody stools, hematemesis	Gastric (cardia) ulcer	NR	Stomach

124	Lalwani et al. (138)<br>Rvw / (29)	2012	India	M	32	Alcoholic liver cirrhosis<br>renal dysfunction	Hematemesis	Gastric (antrum + the greater curvature) ulcer	NR	Stomach
125	Pérez Fernández et al. (139)	2020	Spain	F	71	Nosocomial Pneumonia, MOF, ICU corticosteroids<br>Room adjacent to building under construction	GI bleeding	Gastric ulcer (fundus to antrum) + nodular lesions	NR	Stomach
126	Raju et al. (140)	2020	India	M	48	Diabetes mellitus<br>Gastric adenocarcinoma	Abd pain, nausea, vomiting, dyspepsia, dysphagia, loss of appetite	Gastric (antrum, pylorus) ulcer Eroded distal oesophagus	NR	Stomach
127	Knoop et al. (40)<br>Rvw / (29)	1998	Belgium	M	55	HT/cyclosporine, azathioprine MPD	Abd pain, distension	Gastric (antrum)<br>necrotic ulcers	NR	Stomach,<br>lung (probable)
128	Taams et al. (141)	1992	South Africa	M	26	Blunt abdominal trauma	Peritonitis	NR	NR	Intestine (ileum)
129	Sharma et al. (41)	1998	India	M	0,91	NR	Abd distension, vomiting, peritonitis	NR	NR	Intestine
130	Lalwani et al. (138)<br>Rvw / (29)	2012	India	M	0,75	LT (biliary atresia + biliary cirrhosis) / tacrolimus, MMF	Abd distension	NR	NR	Intestine
131	Lalwani et al.	2012	India	M	35	KT / tacrolimus, MMF, prednisolone	GI bleeding	Cecum ulcer	NR	Intestine
132	Saltmarsh et al. (142)	2014	USA	M	37	AIDS, CD4+, 66 cells/mm3	Abd pain, nausea	NR	Pneumoperitoneum + free fluid	Intestine
133	Ju et al. (143)	2001	South Korea	M	33	KT, MPD<br>acute rejection	NR	Cecum + ascending colon ulcerations, oesophagus erythematous patches	NR	Intestine (colon)
134	Kaneko et al. (144)<br>Rvw / (27)	2018	Japan	F	53	Alcohol + tobacco use<br>Poor nutrition status<br>ICU / septic shock (pneumococcal pneumonia), MV, hemodiafiltration hydrocortisone,	Fever, rectal bleeding	Residual rectum ulcers and bleeding	Pneumoperitoneum	Intestine (colon)
135	Nidhi et al. (145)<br>Rvw / (27)	2019	India	M	2	Malnutrition	Abd pain	NR	Right liver lobe multifocal hypodense lesions, jejunum and ileum wall thickening, right colon hypodense lesion	Intestine (colon)
136	Hyvernat et al. (146)	2020	France	F	52	Crohn disease, corticosteroids and azathioprine<br>Peritonitis/intestinal perforation<br>MOF<br>*A. fumigatus* wound infection	Abd pain, intestinal occlusion	NR	No abnormalities	Intestine (colon)

137	Wotiye et al. (147)<br>Rvw / (27)	2020	India	M	40	Chronic alcohol abuse,<br>Cholecystitis, cholecystectomy<br>Metabolic acidosis, ICU	Abd pain, vomiting, loose stools	Right transverse colon oedematous, thickening + ulcer	Cholecystis, ascites, nodular liver	Intestine (colon)
138	Liu et al. (148)	2024	China	F	65	Diabetes mellitus<br>CD4+ 304 cells/mm3	Nausea/vomiting, diarrhoea, bloody stools	Ileocecal + colon ulcers, hyperaemia, oedema, erosion	Liver hypodense foci, lower intestine wall thickening, enlarged mesenteric lymph nodes	Intestine (colon)
139	Mungazi et al. (149)	2017	Zimbabwe	M	22	History of chronic GI bleeding	Vomiting, hematemesis, melena	Duodenum fungating mass + milliary appearance	NR	Intestine (duodenum)
140	Sun et al. (150)<br>Rvw / (27)	2017	China	M	66	Rheumatic heart disease, mitral stenosis<br>ICU, parenteral nutrition	Abd distension, nausea, upper GI bleeding, haematochezia	Duodenum lesions, exudate	No bleeding	Intestine (duodenum, jejunum)
141	Poyuran et al. (151)<br>Rvw / (27)	2020	India	M	17	Intracardiac surgery<br>ICU, steroid therapy<br>Right hemicolectomy for ileocecal perforation	NR	NR	NR	Intestine (Ileo-caecal)
142	Bhat et al. (152)<br>Rvw / (27)	2022	India	F	22	Puerperal sepsis, uterus surgery<br>ICU	Abd pain, distension, vomiting, constipation<br>Pneumoperitoneum	NR	NR	Intestine (ileo-colic)
143	Taams et al. (141)	1992	South Africa	M	26	Blunt abdominal trauma	Peritonitis	NR	NR	Intestine (ileum)
144	Gupta et al. (153)<br>Rvw / (27)	2018	India	F	1,08	Diabetes, ketoacidosis<br>PICU<br>Perforation peritonitis	Abd distension	NR	NR	Intestine (Ileum)
145	Aruni et al. (154)<br>Rvw / (27)	2020	India	M	49	IV drug abuse, HVC infection<br>Metabolic acidosis<br>Perforation peritonitis	Abd pain, distension, bilious vomiting, constipation	NR	NR	Intestine (ileum)
146	Himaal et al. (155)<br>Rvw / (27)	2021	India	M	52	Diabetes mellitus<br>H1N1 pneumonia, ARDS,<br>ICU, ECMO	Abd distension, melena	Inconclusive	Ileal obstruction, perforation	Intestine (ileum)
147	Manda et al. (156)	2022	India	F	40	KT<br>History of COVID 19, high dose of steroids, ICU	Fever	NR	PET-Scan: right lung lower lobe fibro-cavitary lesion, terminal ileum and caecum perilesional ground glass haziness	Intestine (ileum)
148	Zulpi et al. (157)	2023	India	M	12	Diabetes mellitus, ketoacidosis<br>Post COVID-19 disease	Abd pain, distension<br>Peritonitis	NR	X ray: signs of bowel perforation	Intestine (ileum)
149	Chethan et al. (158)	2012	India	M	31	Chronic renal failure, haemodialysis	Abd pain, black stool	Cecum + ileum ulcerative lesions	Thickened, dilated, fluid-filled, hyperperistaltic bowel loops, shrunken kidneys	Intestine (ileum, cecum)

150	Yadav et al. (159)<br>Rvw /Ozbek et al. 2024	2021	India	F	38	Diabetes mellitus<br>COVID-19 (DG 18 d ago), dexamethasone	Abd pain, vomiting, constipation, massive lower (GI) bleeding.	NR	NR	Intestine (ileum, colon)
151	Sarkardeh et al. (160)	2022	Iran	M	54	Diabetes mellitus<br>COVID-19	NA (pooled data)	NR	NR (pooled data)	Intestine (ileum, colon)
152	Martinello et al. (161)<br>Rvw / (27)	2012	Australia	M	59	Alcohol use, irritable bowel syndrome, malnutrition<br>*Salmonella typhimurium* enteritis<br>Septic shock/peritonitis, MOF, ICU	NA	NR	NR	Intestine (jejunum)
153	Kumar et al. (162)<br>Rvw / (27)	2017	India	F	24	Abd surgery / ileal perforation<br>Mesenteric TB (suspected)	Bilious oozing from the suture line<br>Bilio-purulent fluid from the drain tube	NR	X ray / free gas under the diaphragm	Intestine (jejunum)
154	Budhiraja et al. (163)<br>Rvw / (27)	2019	India	M	0,5	ICU stay 12 d ago	Bilious vomiting, loose stools<br>Abd distension	NR	NR	Intestine (jejunum)
155	Bhana et al. (164)	2023	South Africa	M	29	Abd stab injury, hemoperitoneum<br>ICU, metabolic acidosis	NR	NR	NR	Intestine (jejunum)
156	Watson et al. (165)	2024	Australia	F	63	Digestive surgery for adenocarcinoma	Enteric fluid discharging from laparotomy wound, peritonitis	Gastric +peristomal ulcers	NR	Intestine (jejunum)
157	Paliwal et al. (166)	2022	India	F	17	COVID-19	Abd pain, nausea, vomiting	NR	Enlarged abdominal lymph nodes, thickening of the distal ileal loops and omentum	Intestine (jejunum, ileum)
158	Horowitz et al.	1974	Israel	M	50	NR	Abd pain, vomiting, peritonitis	NR	NR	Intestine (sigmoid colon)
159	Sriperumbuduri et al. (167)<br>Rvw / (27)	2017	India	M	45	Membranous nephropathy, corticosteroids<br>ICU, blood transfusions	Abd distension, vomiting, melena, haematochezia	Proximal ascending colon ulcer	NR	Intestine (sigmoid colon)
160	Singh et al. (168)<br>Rvw / (27)	2021	India	M	48	COVID-19<br>ICU, septic shock, MPD	Abd pain, vomiting, constipation, massive lower (GI) bleeding.	NR	Pneumoperitoneum, colon perforation	Intestine (sigmoid colon)
161	Gupta et al. (153)<br>Rvw / (27)	2018	India	M	2	Sepsis<br>PICU, CAPD	Abd distension, bilious aspirates, bleeding per rectum	NR	Pneumoperitoneum, ascites, enlarged left kidney, renal infarcts, perinephric collection communicating with small bowel	Intestine (small intestine, colon)
162	Sharma et al. (41)	1998	India	M	0,83	NR	Appetite loss, haematochezia, fever, peritonitis	NR	NR	Intestine, cecum

163	Nagy-Agren et al. (169)	1995	USA	F	36	AIDS, CD4+ 70 cells/mm3<br>IV drug use	GI bleeding	NR	NR	Small + large intestine
164	Cebisli et al. (170)	2023	Turkey	F	3	LT, Roux-en-Y hepaticojejunostomy<br>Tacrolimus, MPD<br>Graft rejection, ICU	NR	NR	NR	Small + large intestine
165	Saraf et al. (171)	2024	India	M	51	NR	Abd pain, distension, melena	NR	Cecum thickening + perforation, terminal ileal loops, ascending and transverse colon thickening, air collection	Small + large intestine
166	Eiser et al. (172)	1987	USA	M	57	Haemodialysis, iron overload,<br>Deferoxamine<br>pericarditis, intestinal perforation	NR	NR	NR	Small intestine
167	Yinadsawaphan et al. (173)<br>Rvw / (27)	2020	Thailand	F	25	HIV, CD4+ 419 cells/mm3,<br>Grave's disease<br>*S. pyogenes* toxic shock syndrome,<br>metabolic acidosis, hydrocortisone	Feeding intolerance, bloody diarrhoea, distended abdomen, fever	NR	Pneumoperitoneum, jejunum + ileum perforation	Small intestine
168	Li et al. (174)<br>Rvw / (27)	2019	China	F	67	NR	Hematemesis	Duodenal ulcer (5.5 cm), whitish-yellow exudate, bleeding	Duodenal mass that involved the pancreas head, regional lymph nodes	Oesophagus
169	Evert et al. (175)<br>Rvw / (122)	2021	Germany	M	52	Obesity,<br>COVID 19, ICU, ECMO, dialysis,<br>steroids, tozilizumab	NR	NR	NR	Oesophagus
170	Danion et al. [b] (10)	2022	France	M	64	COVID-19, ICU, MV	NR	NR	NR	Digestive, site NS
171	Marcó del Pont et al. (176)	2000	Argentina	M	2	LT (AHV) + re-transplantation<br>azathioprine, hydrocortisone, MPD,<br>cyclosporine<br>Metabolic acidosis	Abd haemorrhage<br>dark, necrotic surgical wound	NR	NR	Liver
172	Mekeel et al. (177)	2005	USA	F	56	HCV hepatitis<br>Diabetes mellitus<br>KT (10 M)	Mass in epigastrium	NR	NR	Liver
173	Zhan et al. (178)	2008	China	M	41	KT + LT (end stage liver disease+<br>hepatorenal syndrome)<br>Surgery for hepatic artery slit then renal artery endomembrane damage	Abd bleeding	NR	NR	Liver
174	Abboud et al. (179)	2012	Brazil	F	23	LT, MPD, tacrolimus, MMF<br>Hepatic abscess drainage	NR	NR	NR	Liver
175	Chowdhary et al. (180)<br>Rvw / (27)	2020	India	M	57	Diabetes mellitus<br>LT (5 M ago), everolimus, MMF<br>Liver dysfunction	NR	NR	Liver allograft hypodense lesions	Liver

176	Teira et al. (181)<br>Rvw / (182)	1994	Spain	M	33	AIDS, CD4+ 57 cells/mm3<br>Self-limited pancreatitis (3M ago)	Epigastric discomfort, fever	NR	Spleen fluid areas (4 and 7 cm in diameter)	Spleen
177	Chakrabarti et al. (183)	2003	India	M	45	Back trauma 5 M ago	Flanks pain, dysuria, pyuria	NR	Spleen abscesses, spleen and kidneys infarcts	Spleen
178	Gupta et al. (184)	2010	India	M	28	NR	Flank pain, anorexia, fever	NR	Enlarged left kidney with infarcts, perinephric inflammation, splenic infarct, peri-splenic collection	Spleen
179	O'Connor et al. (185)	2018	Ireland	F	36	Double lung transplant	Massive haematemesis + melaena		NR	Spleen
180	Luo et al. (182)	2022	China	F	68	Diabetes mellitus, ketoacidosis	Abd pain		Spleen patchy hypodense shadow, wedge-shaped changes	Spleen
181	Jain et al. (186)<br>Rvw / (27)	2021	India	F	57	Diabetes COVID 19 (DG 20 d ago), PO MPD	Abd pain, distension, constipation	NR	Terminal ileum and caecum thickening;<br>Peritoneal and retroperitoneal fat in right iliac fossa and pelvis inflammation	Mesenteron
182	Okhuysen et al. (187)	1994	USA	M	29	Traffic accident<br>Multiple injuries with right-flank soft-tissue crush injury	Necrotic right-flank wound+ induration, erythema.	NR	Extensive loss of soft tissue and bone	Intra-abdominal
183	Durila et al. (188)	2016	Czech Republic	F	12	Hepatitis VHA, ascites drainage iatrogenic spleen punction, laparotomies for intra-abdominal bleeding	NR	NR	NR	Intra-abdominal
184	Khanna et al. (129)<br>Rvw / (189)	1980	USA	M	NR	Diabetes mellitus, CAPD	NR	NR	NR	Peritoneal
185	Polo et al. (190)<br>Rvw / (189)	1989	Spain	M	48	CKD, CAPD<br>Bath in a farm cistern	Abd pain, fever, cloudy peritoneal fluid	NR	NR	Peritoneal
186	Fergie et al. (191)<br>Rvw / (189)	1992	USA	F	0,13	Down's syndrome, pulmonary artery banding<br>ICU, acidosis, CAPD	Cloudy peritoneal fluid<br>Abd distension, ileus	NR	NR	Peritoneal
187	Adam et al. (192)<br>Rvw / (189)	1994	USA	M	47	SLE, chronic renal failure<br>CAPD	NR	NR	NR	Peritoneal
188	Khan et al. (193)<br>Rvw / (189)	2000	Kowait	M	65	Peritoneal dialysis	NR	NR	NR	Peritoneal
189	Monecke et al. (194)<br>Rvw / (189)	2006	Germany	F	62	KT, MPD + cyclosporine<br>ICU, MOF *C. glabrata* peritonitis after sigmoid colon perforation	Mould-like plaques on the peritoneum, on Dacron mesh	NR	NR	Peritoneal

190	Pimentel et al. (195)<br>Rvw / (189)	2006	Australia	F	39	Diabetes mellitus, CAPD	Abd pain, vomiting, diarrhoea, cloudy PD bag	NR	Abd free fluid	Peritoneal
191	Nayak et al. (196)<br>Rvw / (189)	2007	India	M	62	CAPD, breach in sterile technique	Peritonitis	NR	NR	Peritoneal
192	Bhutada et al. (197)	2012	India	M	52	CAPD after KT rejection	Abd pain, vomiting, fever	NR	NR	Peritoneal
193	Pamidimukkala et al. (198)	2020	India	M	45	Chronic Kidney Disease, CAPD<br>History of bacterial peritonitis episodes	Abd pain, nausea, discoloration and ulceration of the skin and soft tissue surrounding the site of CAPD insertion	NR	NR	Peritoneal
194	Dalgic et al. (199)	2011	Turkey	M	14	Papillon–Lefevre syndrome	Abd pain, distension, ascites	NR	Left liver semisolid masses with cystic component with irregular septae, ascites	Peritoneal,<br>liver (possible)
195	Adam et al. (192)	1994	USA	F	36	Abd surgery	NR	NR	NR	Abd wall (skin, muscle)
196	Verma et al. (200)<br>Rvw / (201)	1995	India	M	45	Trauma, fall from height	NR	NR	NR	Abd wall
197	Chakrabarti et al. (202)<br>Rvw / (201)	1997	USA	M	70	Diabetes mellitus,<br>Trans-vesical prostatectomy	Swelling, blackish discoloration around the wound, necrotizing fasciitis	NR	NR	Abd wall
198	Mathews et al. (203)<br>Rvw / (201)	1997	India	F	32	Caesarean delivery	Pain, swelling around the caesarean scar, fever, necrotizing fasciitis	NR	NR	Abd wall
199	Kumar et al. (204)<br>Rvw / (201)	2003	India	M	50	NR	Fever, boil in the right upper anterior abdominal wall then necrotizing fasciitis + cotton wool-like material	NR	NR	Abd wall
200	Thami et al. (205)<br>Rvw / (201)	2003	India	M	28	Appendicectomy	Ulcerated, painful operative scar, necrotizing fasciitis + cotton wool-like material	NR	NR	Abd wall
201	Kerbaul et al. (206)	2004	France	M	61	HT/NS immunosuppression, corticoids	Black necrotic skin lesion	NR	NR	Abd wall
202	Padmaja et al. (207)<br>Rvw / (201)	2006	India	M	35	Appendicectomy	Abd pain, fever, gangrenous operative scar, necrotizing fasciitis + cotton wool-like material	NR	NR	Abd wall
203	Belfiori et al. (208)	2007	Italy	M	17	Accident with agricultural machinery (soil contamination)<br>Abd wall, IA surgery, ICU	Vomiting	NR	NR	Abd wall
204	Tilak et al. (209)<br>Rvw / (201)	2009	India	F	26	Surgery for ovarian cystectomy, subsequent cellulitis	Wound necrosis, fever, necrotizing fasciitis +cotton wool-like material	NR	NR	Abd wall

205	Tilak et al. (209)<br>Rvw / (201)	2009	India	F	24	Caesarean delivery	Wound necrosis, fever, necrotizing fasciitis +cotton wool-like material	NR	NR	Abd wall
206	Tapish et al. (210)<br>Rvw /(201)	2010	India	F	48	Abd spider bite	Abd wall swelling, pain, fever, then necrotizing fasciitis	NR	NR	Abd wall
207	Alharbi et al. (211)<br>Rvw / (27)	2017	Canada	M	31	Diabetes mellitus CAPD	Abd pain, fluctuant induration (15x7 cm) / catheter site, purulent drainage	NR	No evidence of intra-abdominal infection	Abd wall
208	Pamidimukkala et al. (198)	2020	India	M	27	Abd surgery (inguinal herniorrhaphy)	Necrotizing wound infection + lower abdominal wall ulceration	NR	NR	Abd wall
209	Pamidimukkala et al.	2020	India	M	35	Appendicectomy	Necrotizing wound infection, painful swelling	NR	NR	Abd wall (fasciitis)
210	Pamidimukkala et al.	2020	India	F	23	Tubectomy	Necrotizing wound infection	NR	NR	Abd wall (fasciitis)
211	Pamidimukkala et al.	2020	India	F	30	Appendicectomy	Necrotizing wound infection	NR	NR	Abd wall (fasciitis) xiphisternum to the suprapubic region, between flanks
212	Pamidimukkala et al.	2020	India	M	50	Chronic alcohol use<br>Traffic accident, chest and abdominal walls injuries	Chest and Abd walls blackish discoloration + ulceration extended to muscle layers, paralytic ileus, renal failure	NR	NR	Abd wall, omentum, intestine, kidney, chest wall
213	Taams et al. (141)	1992	South Africa	M	68	Thoraco-abdominal stab wounds<br>Bowel perforations, abdominal surgery<br>Septicaemia	NR	NR	NR	Abd wall, intestine (ileum, colon)
214	Taams et al.	1992	South Africa	M	38	Left lower chest stab wound	Peritonitis, haematuria	NR	NR	Abd wall, intestine (ileum, colon)
215	Ram et al. (212)<br>Rvw / (189, 201)	2007	India	M	59	Diabetes mellitus, CAPD<br>Coagulase negative staphylococcal peritoneal infection	Abd pain, cloudy dialysis effluent erythematous, necrotic Abd wall exposing muscles	NR	NR	Abd wall, peritoneal
216	Patel et al. (213)<br>Rvw / (201)	2010	India	M	75	Diabetes mellitus<br>Abdominal surgery for mesenteric vascular insufficiency	Surgical site black margins + erythema + skin wall oedema	NR	NR	Abd wall, intestine (colon)
217	Zhao et al. (214)	2012	China	M	57	KT + LT (10 Y later)<br>tacrolimus, MMF, prednisone	Exudates then necrosis inside the transplant wound	NR	NR	Abd wall, kidney
218	Paonam et al. (215)	2014	India	F	21	Percutaneous drainage of renal collection	Bilateral flank pain, dysuria, oliguria, pyuria, fever	NR	Renal, perirenal collections	Abd wall, kidneys

219	Narayanaswamy et al. (216)	2023	India	M	30	Chronic alcohol + cannabis use<br>Malnutrition	Painful, erythematous, oedematous left flank	NR	Bulky hypodense left kidney + air foci, perinephric collection (fascia, retroperitoneal space, subcutaneous space), splenic air foci	Abd wall, intestine (colon), spleen, kidney, retroperitoneal (probable)
220	Stein et al. (217)<br>Rvw / (218)	1965	South Africa	M	35	Anaemia	Peritonitis	NR	NR	Stomach, peritoneal
221	Lawson et al. (34)	1974	South Africa	F	22	Acute renal failure	Acute renal failure	NR	NR	Oesophagus, stomach, intestine (colon)
222	Lawson et al.	1974	South Africa	M	50	Mental confusion, diarrhoea	Mental confusion, diarrhoea	NR	NR	Stomach, (intestine) colon
223	Eiser et al. (172)	1987	USA	M	45	History of 2 KT rejection, haemodialysis, iron overload, Deferoxamine	Jaundice, ascites, hepatic coma	NR	NR	Liver, intestine (jejunum, ileum)
224	Branton et al. (219)<br>Rvw / (189)	1991	USA	M	61	CAPD, multiple episodes of peritonitis	Clogged Tenckhoff catheter<br>Abd pain, distension, fever	NR	No evidence of intra-abdominal abscess	Intra-abdominal abscess, ileal wall
225	Nordén et al. (220)	1991	Sweden	M	40	KT, cyclosporine, azathioprine, steroids	Surgical wound serous secretion then necrosis, graft dysfunction	NR	NR	Intra-abdominal, pelvic, thoracic
226	Taams et al. (141)	1992	South Africa	F	30	Thoracoabdominal stab, peritonitis, pneumothorax	Peritonitis	NR	NR	Intestine (colon), omentum
227	Vadeboncoeur et al. (221)	1994	Canada	F	15	Septic shock, meningococcaemia	Rectum mucosal sloughing then haemorrhagic colitis	NR	NR	Intestine (colon) then intra-abdominal abscess
228	Hosseini et al. (222)	1998	USA	F	42	SLE, prednisone<br>ICU	Peritonitis	Sigmoid colon haemorrhagic mucosa with erosion, swelling	NR	Stomach, small and large intestine
229	Guardia et al. (223)	2000	USA	M	42	IV drug use<br>AIDS CD4+ 4cells/mm3	Flank pain, dysuria, macroscopic haematuria, nausea, vomiting	NR	Enlarged kidneys	Kidney, peripancreatic tissue
230	Herbrecht et al. (224)	2001	France	M	78	LT, tacrolimus, steroid	NR	NR	NR	Oesophagus, stomach, mediastinum
231	Nannini et al. (225)<br>Rvw / (189)	2003	USA	M	43	End-stage renal disease,<br>Previous failed KT, CAPD<br>episodes of bacterial peritonitis	Abd pain, fever	NR	KT cystic lesion<br>Follow up (w 6 of ATFT): pelvic collection	Peritoneal, intra-abdominal abscess
232	Serna et al. (226)<br>Rvw / (189)	2003	USA	M	42	KT chronic rejection, CAPD<br>KT lymphocele or urinoma,<br>E. coli peritoneal infection	Abd pain, cloudy dialysis effluent	NR	KT lymphocele or urinoma, abscess in the area of the KT<br>Follow up: persistent intra-abdominal abscesses	Peritoneal, intra-abdominal abscess
233	Alkhunaizi et al. (227)	2005	Saudi Arabia	M	41	KT, transplant tourism PakistanAcute rejection, monoclonal Ab OKT3	Oliguria, delayed graft function	NR	Lesions in the liver, lungs, and spleen	Kidney, liver

234	Sethi et al. (228)	2018	India	F	17	Idiopathic CD4+ lymphocytopenia (250 cells/mm3)<br>Liver cirrhosis<br>PO prednisone	Right flank tenderness.	NR	Right renal artery thrombosis, perinephric collection with extension into the liver	Liver, kidney
235	Deja et al. (229)<br>Rvw / (29, 38, 230)	2006	Germany	M	48	Traffic accident, gastric tube decreased HLA-DR expression on monocytes of positive titre for parietal cell antibodies	NR	Gastric, ulcer and necrosis, coecum, colon ulcers	NR	Stomach, intestine (coecum, colic)
236	Manchikalapati et al. (231)<br>Rvw / (38)	2006	USA	M	41	HLT / pulmonary arterial hypertension<br>PD, MMF, monoclonal antibody	Abd pain, distension, gastric bleeding, melena	Gastro-oesophageal junction ulcer, then gastric necrotic ulcer, exudate; duodenal bulb ulcers	Gastric fundus mass 4.1 × 3.8-cm, splenic infarcts, omentum fat-containing masses	Oesophagus, stomach, intestine (duodenum, ileum, cecum)
237	Jung et al. (232)<br>Rvw / (29)	2007	South Korea	M	43	Diabetes mellitus<br>Alcohol use, chronic pancreatitis, endoscopic retrograde cholangiopancreatography 8 M ago	Abd pain	NR	Gastric wall thickening, dirty air bubbles, pneumoperitoneum	Stomach, liver, Abd muscle layer and serosa
238	Sedlacek et al. (189)	2008	Lebanon	F	57	Polycystic kidney disease, CAPD<br>Peritonitis resolved under antibiotic treatment	Abd pain, dialysate cloudiness, fever	NR	Unremarkable<br>1st follow up: pelvic abscess<br>2nd follow up: peritoneal nodules	Peritoneal, intra-abdominal abscess
239	Berne et al. (233)<br>Rvw / (29)	2009	USA	M	55	Traffic accident, Abd surgery	Abd pain, fever	NR	No clear abnormalities	Stomach, Abd wall
240	Islam et al. (234)<br>Rvw / (29)	2009	South Africa	M	NR	Abd surgery for peritonitis (necrotic small bowel, sigmoid colon)	Abd pain, distension, vomiting, constipation	NR	NR	Stomach, spleen
241	Mezhir et al. (235)	2009	USA	M	50	Poly substance abuse,<br>HCV hepatitis, haemodialysis<br>ICU, MOF, sepsis<br>Abdominal surgery /colic perforation	GI haemorrhage	Necrotic lower oesophageal + gastric ulcers	Liver right lobe cystic lesion	Stomach, liver (d 35 after stomach)
242	Van Sickels et al. (236)	2011	USA	M	26	Chest and abdomen gunshot wounds, surgery<br>Gastric, diaphragmatic, liver injuries	thick purulent drainage: posterior to the stomach; left side of the liver	NR	NR	IA, liver
243	Rudler et al. (237)<br>Rvw / (38)	2012	England	M	58	ICU, septic shock, MOF, MV	GI haemorrhage	Gastric ulcer, bleeding	NR	Stomach, intestine (colic), spleen
244	Tan et al. (238)	2012	Brunei	F	54	Diabetes mellitus, haemodialysis, history of CAPD, gastric erosions	Abd pain	NR	Small-bowel obstruction, thickened peritoneum, loculated fluid collections	IA abscess, peritoneal
245	Enani et al. (239)<br>Rvw / (29)	2014	Saudi Arabia	M	54	Diabetes mellitus, chronic kidney disease	Abd distension, melena	Gastric ulcer	Perforation of posterior wall of the stomach air in the abdomen, spleen infarction	Stomach, spleen

246	Gaut et al. (240)<br>Rvw / (27, 29)	2017	USA	F	48	Diabetes mellitus,<br>HVC-induced cirrhosis<br>LT/MMF, Tacrolimus, prednisone<br>portal gastropathy	Abd pain, distension	NR	Ischemic right colon, retroperitoneal foci of air	Retroperitoneal, small and large intestine, stomach, liver, pancreas, peripancreatic fat, bladder, femoral artery and vein
247	Gupta et al. (153)<br>Rvw / (27)	2018	India	F	1,5	Hypotensive shock, PICU<br>metabolic acidosis	Fever, diarrhoea, vomiting	NR	NR	Oesophagus, stomach, intestine (colon)
248	Izaguirre-Anariba et al. (241)<br>Rvw / (27, 29)	2018	USA	M	57	Alcohol abuse,<br>stage-IV sarcoidosis / long-term oral prednisone	Hematemesis	Gastric ulceration	Bladder wall thickening hydroureter, hydronephrosis	Stomach, bladder
249	Aggarwal et al. (242)<br>Rvw / (27)	2019	USA	M	70	LT, tacrolimus, MMF, MPD	Melena, haematochezia	Colon (25 cm from the anal verge) inflammatory mass, cratering ulceration	Sigmoid colon chronic walled-off perforation	Intestine (colon),<br>IA abscess
250	Reis et al. (243)<br>Rvw / (27)	2019	Brazil	F	25	Lung T (cystic fibrosis) / MPD, cyclosporine, MMF and prednisolone<br>Lung graft dysfunction<br>Renal dysfunction, metabolic acidosis	Abd distension, pain	NR	Hepatic abscess, colon increased densification	Intestine (ileum, colon), liver, lymph nodes
251	Rotundo et al. (244)<br>Rvw / (29)	2019	USA	M	24	Testicular cancer, cycle 2 of cisplatin/ etoposide<br>ICU, MV	Melena	Gastric transmural necrosis (90%)	gastric intramural emphysema	Stomach, Abd cavity
252	Sehmbey et al. (245)<br>Rvw / (27)	2019	USA	M	47	Trans-pelvic gunshot<br>Abdominal surgery	Upper GI bleeding	Gastric (body, fundus) ulcer, exudate	NR	Stomach, small intestine anastomosis, rectal stump
253	Shankaralingappa et al. (246)	2019	India	F	69	Malnutrition, iron injection<br>Pancolitis (unknown origin), steroids	Abd distension, vomiting,	NR	Pneumoperitoneum	Intestine (ileum, colon), mesenteron
254	Pamidimukkala et al. (198)	2020	India	F	29	Traffic accident, degloving injury gluteal regions extending<br>to the perineum, rectum, anal canal	Necrotizing wound infection	NR	NR	Perineum, intestine (rectum, anal canal)
255	Petrochko et al. (247)<br>Rvw / (27)	2020	USA	M	70	Diabetes mellitus LT /MPD, tacrolimus, MMF<br>Temporary abdominal closure<br>ICU, hepatic congestion, haemodialysis, tracheostomy / MV, parenteral nutrition	Bowel movements, bleeding	Sigmoid colon ulceration	Colonic diverticulosis	Intestine (sigmoid colon), Abd subcutaneous tissue
256	Shah et al. (248)	2020	USA	M	72	Diabetes mellitus, gout, splenectomy<br>*S. infantarius* (formerly bovis) septicemia, endocarditis<br>Stress dose corticosteroids<br>Hemodialysis	Melanotic stools then haematochezia	Sigmoid colon erosions, erythema, granularity	Gastric ulcers, Colonic wall thickening<br>then IA + pelvic abscesses	Intestine (colon),<br>IA and pelvic abscess

257	Chiang et al. (249)<br>Rvw / (27)	2021	Taiwan	M	28	Diabetes Mellitus, ketoacidosis<br>Chronic alcohol abuse, pancreatitis.<br>ICU, MOF	Haematochezia	Transverse colon ulcer + mucus, bleeding	NR	Intestine (colon)<br>IA abscess
258	Hammami et al. (250)<br>Rvw / (27)	2021	Tunisia	F	38	Postpartum haemorrhage, hysterectomy	Fever		Gastric (greater curvature) perforation, splenic necrosis, left subdiaphragmatic and pelvic collections	Stomach, spleen
259	Martin-Blais et al. (251)<br>Rvw / (27)	2021	USA	F	9	Multi organ T (liver, pancreas, small bowel, transverse colon)<br>Abd wall closure on post-transplant d 3 /Tacrolimus, MPD, MMF<br>ICU	Fever, Abd pain and distention, vomiting	Inconclusive	Inconclusive	Intestine (ileum, colon), kidney
260	Varshney et al. (252)<br>Rvw / (27)	2021	India	M	35	COVID-19 (DG, 1 M ago)<br>ICU, injectable steroids, MV<br>PO steroids (auto medication) at home<br>New admission in ICU for urosepsis (bilateral renal calculi, double 'J' stent)	Abd pain, melena	NR	Suggestive of pyelonephritis.	Kidney, mesenteron
261	Danion et al. [b] (10)	2022	France	M	60	COVID-19, ICU, MV<br>Stercoral peritonitis	Abd distension	NR	Colon perforation	Intestine (colon), peritoneal
262	Kyuno et al. (253)<br>Ito et al. (254)<br>Same patient<br>Rvw / (122)	2022	Japan	M	58	Previous COVID 19 (27 d)<br>ICU, ARDS, ECMO, glucocorticoids<br>hemicolectomy for intra-abdominal bleeding	Necrotic surgical wound	NR	Intra-abdominal bleed (mesentery of the ascending colon)	IA: intestinal mesentery, + abdominal skin, stoma, wall muscles, spleen, stomach, bladder, kidney
263	Meshram et al. (255)<br>Rvw / (122)	2022	India	M	41	KT, steroid, MMF, tacrolimus<br>Post-transplant diabetes mellitus<br>COVID 19, dexamethasone	Fever, dyspnoea	NR	Splenic abscess/sonography	Spleen, stomach
264	Ralaizanaka et al. (256)	2022	Madagascar	M	75	Diabetes mellitus, alcohol abuse, alcoholic pancreatitis,<br>history of pulmonary tuberculosis	Haematochezia, peritonitis, haemorrhagic shock	Gastric (greater curvature) 3- 4 cm ulcers, budding oedematous, fragile mucosa, rectum fistulous orifice	Rectum anterior wall perforation	Stomach, intestine (rectum)
265	Banerjee et al. (257)	2023	India	F	38	Diabetes mellitus, ketoacidosis<br><br>COVID19 (DG 2 w ago)	Abd pain, nausea, vomiting	NR	Pneumoperitoneum, ileocolic artery thrombosis, cecum, ascending colon, part of the transverse colon gangrene, right kidney infarct	Intestine, mesenteron
266	Rathi et al. (258)<br>Rvw / (29)	2023	India	M	42	Diabetes<br>Alcohol use	Abd pain, vomiting, then hematemesis, then melena	Gastric ulcer (diffuse in the body, antrum, pylorus) then perforation, pancreatic necrosis, gastrocolic fistula	Emphysematous gastritis	Stomach, intestine

267	Li et al. (259)	2010	China	M	36	Previous (5M) adrenal gland fungal infection, ITZ 1M<br>HBV infection	Abd pain, icterus	NR	Hepatic lesion	Adrenal gland then (5M) liver
268	Rabin et al. (260)<br>Rvw / (261)	1961	USA	M	22	Burn 64% body surface	Facial cutaneous necrosis	NR	NR	Disseminated: hearth, lungs, thyroid, kidneys, stomach
269	Straatsma et al. (262)<br>Rvw / (261)	1962	USA	M	28	COPD, corticosteroids	NR	NR	NR	Disseminated: hearth, lung, thyroid, liver, spleen, GI, kidneys
270	Straatsma et al.	1962	USA	M	62	Diabetes mellitus	NR	NR	NR	Disseminated: hearth, lung, kidney, spleen
271	Straatsma et al.	1962	USA	M	28	3rd degree burn, gangrene finger	NR	NR	NR	Disseminated: hearth, lungs, GI, liver, spleen, kidneys
272	Agger et al. (263)	1978	USA	M	37	Alcohol abuse,<br>Abd surgery (haemorrhagic pancreatitis)<br>ICU, CAPD, MV	NR	NR	NR	Disseminated: lung, gastrointestinal
273	Virmani et al. (261)	1982	USA	M	65	Sideroblastic anaemia, hemosiderosis, multiples transfusions	Fever, pneumonia, myocarditis, hepatomegaly	NR	NR	Disseminated: hearth, lung, liver, kidneys, spleen, adrenal, brain
274	Virmani et al.	1982	USA	M	26	Booby trap injuries (shrapnel wounds), legs amputation for extensive necrosis.	Cardiac tamponade	NR	NR	Disseminated: hearth, lung, kidneys, gastrointestinal, skin
275	Gupta et al. (264)	1987	India	M	53	Tobacco use, emphysema<br>Chronic Kidney Disease, CAPD	Vomiting for 15 days, stools with fresh blood, oliguria then anuria<br>Peritonitis	Oesophagitis and gastric ulcers	NR	Disseminated: intestine, mesentery, peritoneum, spleen, kidneys, lung
276	Nakamura et al. (265)<br>Rvw / (189)	1989	USA	M	17	CAPD, history of rejected KT	Nausea, vomiting	NR	NR	Disseminated: peritoneal, spleen, brain, lung, heart
277	Nagy-Agren et al. (169)	1995	USA	M	45	AIDS, CD4+ 26 cells/mm3,<br>IV drug use<br>*C. difficile* colitis	Renal failure, fever	NR	NR, USG: enlarged kidney and liver, splenic collection	Disseminated: liver, spleen, kidney, thyroid, bone marrow
278	Singh et al. (266)<br>Rvw / (29)	1995	USA	M	28	LT, retransplantation<br>Tacrolimus, MPD	NR	Gastric (gastroesophageal junction to cardia) greyish-black exudate	NR	Disseminated: oesophagus, stomach, lung, hearth, aorta

279	Stoebner et al. (267)	2000	France	M	63	KT, prednisone, azathioprine<br>renal vein anastomotic disruption<br>Rhinofacial mucormycosis	MOF	NR	NR	Disseminated: rhinofacial, liver, kidney
280	Tsaousis et al. (268)	2000	Greece	F	73	Diabetes mellitus, obesity	Fever, hepatomegaly	Oesophagitis, multiple diverticulosis of the large bowel	USG, MRI: multiple hepatic lesions	Disseminated: liver, brain (probable)
281	Sehgal et al. (269)	2004	India	M	57	KT, cyclosporine<br>acute rejection, MPD + MMF	Peritonitis, pneumoperitoneum	NR	NR	Disseminated: rhino cerebral (1st site), intestine (colon), peritoneal
282	Gimeno-Garcia et al. (270)<br>Rvw / (29)	2006	Spain	F	73	CAPD methyl-prednisolone,<br>ICU	GI bleeding	Gastric (fundus, body) necrotic areas, ulcers, greenish and yellowish exudates	NR	Disseminated: stomach, pancreas, lung
283	Alexander et al. (271)	2010	USA	M	61	KT, tacrolimus, prednisone<br>same donor than another case	NR	NR	Air and fluid in the explanted kidney bed; lesions in the spleen and liver; thrombosed left common femoral vein; bilateral pulmonary opacities.	Disseminated: intestines, spleen, liver, kidney, lung, heart
284	Arena et al. (272)	2011	Italy	M	32	Hemophagocytic syndrome<br>dexamethasone, cyclosporin, etoposide	Hepatosplenomegaly, fever	Normal	NR	Disseminated: intestine (stomach, jejunum), kidneys, lung
285	Gurevich et al. (273)	2012	Israel	M	10	LT, tacrolimus, steroid,<br>abdominal surgery for bilh leak<br>Ascites infection (*E. coli* and *C. krusei*)	Fever, ascites	NR	Liver abscesses with extension to gastric wall and diaphragm, splenic infarcts, pericardial effusion	Disseminated: liver, pericardial fluid
286	Nam et al. (274)	2015	South Korea	F	45	KT, history of graft rejections;<br>tacrolimus, prednisolone, MMF, rituximab<br>New-onset diabetes, leukopenia (leucocyte nb. NR) episodes	*Paralytic ileus*	NR	Lung cavitary lesions<br>Peri- allograft, splenic abscess, increasing size then pericardial abscess	Disseminated: lung, peritoneal, spleen, brain, heart
287	Alqhamdi et al. (275)<br>Rvw / (250)	2019	Saudi Arabia	M	36	Diabetes mellitus	Diarrhoea for 1 M	Gross spleen invading fundus of the stomach	Left upper lobe air space consolidation<br>Sub-pleural abscess (11 × 10 × 12 cm) invading pleural and splenic, stomach posterior fundus, splenic infarction	Disseminated: lung, spleen

288	Prohaska et al. Rvw / (27)	2020	Germany	M	67	High-dose steroid therapy<br>ICU, ARDS, ECMO, MOF	NR	NR	NR	Disseminated: stomach, intestine (colon), pancreas, kidney, lung, myocardium
289	Evert et al. (175)	2021	Germany	F	44	Obesity, liver steatosis<br>COVID 19<br>ICU, ECMO, dialysis, steroids	NR	NR	NR	Disseminated: heart, intestine
290	Sami et al. (276)<br>Rvw / (122)	2022	Banglades h	M	58	Previous COVID-19, chronic hepatitis	Abd distension, ascites, weight loss	NR	Hepatosplenomegaly, ascites, abdominal lymphadenopathy	Disseminated: Bone marrow, ascites

Table S1. Table S1. Summary of epidemiological and clinical data reported in the 290 cases of gastrointestinal (GI) and intra-abdominal (IA) mucormycosis that were included in the study.

Rvw, reviewed by. M, male. F, female. NR, not reported. AIDS, acquired immunodeficiency syndrome, CD4+, CD4 + lymphocytes, ICU, intensive care unit. PICU, paediatric intensive care unit. Abd, abdominal. KT, kidney transplant. HLT, hearth and liver transplant. LT, liver transplant. HT, hearth transplant; IV, intra-venous. MPD, methylprednisolone. MMF, mofetil mycophenolate. COPD, chronic obstructive pulmonary disease. SLE, systemic lupus erythematosus. ANCA, anti-neutrophilic cytoplasmic autoantibody, ARDS, acute respiratory distress syndrome. MOF, multi-organ failure. CAPD, continuous ambulatory peritoneal dialysis. MV, mechanical ventilation. HAV, hepatitis A virus. HBV. HCV, hepatitis C virus. pmn, polymorphonuclear. DG, diagnosis. d, day. W, week. M, month.

a. Outbreak/wooden tongue depressors
b. Additional personal data

	References	Infection site	Diagnosis				Treatment		Outcome at3 M post DG
			**Histopathology** *Mucorales* type hyphae	**Mycology, Molecular DG**	**Fungal agent identification**	**Concomitant infection**	**AFT dosage, duration (d, w, M)**	**Surgery / other**	
1	Sutehreland et al.	Stomach	Autopsy: stomach	None	NI	Pulmonary TB Liver, lung schistosomiasis	None (post-mortem diagnosis)	None	Dead, post-mortem diagnosis
2	Kahn et al.	Stomach	Autopsy: stomach	None	NI	*S. aureus* septicaemia	None (post-mortem diagnosis)	None	Dead 7 d before DG, post-mortem diagnosis
3	Abramowitz et al.	Stomach	Autopsy: stomach	None	NI	NR	None	None	Dead, post-mortem diagnosis
4	Dannheimer et al.	Stomach	Autopsy: stomach	NR	NI	Cerebral cysticercosis and a haemorrhagic bilharzial cystitis	None (postmotem DG)	None	Dead
5	Lawson et al.	Stomach	Gastric sample	None	NI	NR	None	Laparotomy	Dead during surgery
6	Lawson et al.	Stomach	Gastric sample	None	NI	NR	None	Gastrectomy	Dead
7	Lawson et al.	Stomach	Gastric sample	None	NI	NR	None	Ulcer suturation	Dead
8	Lawson et al.	Stomach	Autopsy: stomach	None	NI	NR	None	None	Dead, post-mortem diagnosis
9	Lawson et al.	Stomach	Gastric sample, No vascular invasion	None	NI	NR	None	Partial gastrectomy/ulcer resection	Alive
10	Lawson et al.	Stomach	Gastric sample, No vascular invasion	None	NI	NR	None	Ulcer suture	Alive
11	Shulmnan et al.	Stomach	Gastric Bx	None	NI	NR	IV AMB	None	Alive
12	Brullet et al.	Stomach	Gastric Bx	Bx Negative culture	NI	*HIV, Pneumocystis jirovecii, HHV8 (Kaposi sarcoma), Oral candidiasis*	KTZ, 3 w	None	Alive
13	Winkler et al.	Stomach	Gastric Bx	Positive DE + culture	*Rhizopus arrhizus* (formerly *oryzae), Rhizopus stolonifer*	GI CMV infection Gastric *C. glabrata*	IV AMB 1mg/kg/d	None	Alive

14	Corley et al.	Stomach	Gastric Bx	Positive culture	Rhizopus sp.	NR	IV AMB	Partial gastrectomy	Alive /cured
15	Knoop et al.	Stomach	Gastric sample	Positive culture	*Rhizopus microsporus* var. *rhizopodiformis*	NR	IV LC AMB, 3 mg/kg/d	Superior polar gastrectomy	Dead at 2 M
16	Sharma et al.	Stomach	Gastric sample	None	NI	NR	None	Gastrectomy	Dead
17	Sheu et al.	Stomach	Gastric Bx	Negative culture/Bx	NI	NR	IV AMB	None	Dead
18	Barosso et al.	Stomach	Gastric Bx	None	NI	NR	IV L AMB	None	Dead, 3 w after admission
19	Cherney et al.	Stomach	Gastric sample (antemortem)	None	NI	Peritonitis *Yersinia* sp, *Enterobacter* sp, *Enterococcus* sp., *Candida krusei*	None (post-mortem diagnosis)	Gastrectomy	Dead, post-mortem diagnosis
20	Al-Rikabi et al.	Stomach	Gastric sample	Positive culture	NS	Fungal (NS) septicemia	None	Partial gastrectomy	Died
21	Pickeral et al.	Stomach	Gastric brushing, Bx	None	NI	*Clostridium difficile* colitis	IV LC AMB, 1 mg/kg/d	None	Dead, 3 d post-diagnosis
22	Geramizadeh et al.	Stomach	Autopsy: stomach	None	NI	NR	None	None	Dead
23	Tinmouth et al.	Stomach	Gastric sample	Positive culture/gastric sample	*R. microsporus* var. *rhizopodiformis*	Pulmonary: *Aspergillus fumigatus*	IV AMB	Spleen flexure, gastric fundus, diaphragm, descending colon debridement	Dead, 8 d after surgery Post-mortem diagnosis
24	De Oliveira et al.	Stomach	Gastric sample	None	NI	NR	IV AMB	None	Dead 32 d after admission
25	Park et al.	Stomach	Gastric Bx	Positive culture	*Rhizopus* sp.	NR	None	Proximal gastrectomy	Alive
26	Shahapure et al.	Stomach	Gastric Bx	None	NI	NR	IV AMB	Partial gastrectomy	Alive
27	Vera et al.	Stomach	Gastric sample	None	NI	NR	IV L AMB 4 mg/kg/d, 6 w	Partial gastrotomy	Alive
28	Maravi-Poma et al.	Stomach	Gastric Bx	Positive culture / gastric sample	*R. microsporus* var. *rhizopodiformis*	NR	IV AMB 1 mg/kg/d, total dose 1120 mg	Gastrectomy	Dead

29	Maravi-Poma et al.	Stomach	Gastric Bx	None	*Rhizopus* sp.	NR	IV AMB 1 mg/kg/d, total dose: 140 mg	None	Dead
30	Maravi-Poma et al.	Stomach	Gastric Bx	Positive culture/ gastric fluid	*Rhizopus* sp.	NR	IV AMB 1 mg/kg/d, total dose: 2795 mg	None	Alive
31	Maravi-Poma et al.	Stomach	Gastric Bx	Positive culture / gastric Bx, gastric fluid	*R. microsporus* var. *rhizopodiformis*	NR	IV AMB 1 mg/kg/d, total dose: 2772mg	Gastrectomy	Dead, postoperative bacterial septic shock
32	Maravi-Poma et al.	Stomach	Autopsy: stomach	None	NI	NR	None (post-mortem diagnosis)	None	Dead, post-mortem diagnosis
33	Stamm et al.	Stomach	Gastric sample Positive Immunostaining with anti-*Rhizomucor*/*Rhizopus* Abs	None	*Rhizomucor* sp. / *Rhizopus* sp.	NR	None	4/5 gastrectomy	Alive
34	Prasad N. et al.	Stomach	Gastric Bx then sample	None	NI	Gastric: *Candida, Strongyloides. Stercolaris* Septicemia : *Klebsiella pneumoniae* +*Pseudomonas aeruginosa*	IV L AMB 50 mg/d, total dose 3g	Distal gastrectomy	Alive
35	Devlin et al.	Stomach	Gastric sample	Positive DE and culture	*Rhizopus* sp.	*C. albicans*, *E. faecium*, and *Stenotrophomonas maltophila*	IV LC AMB, 350 mg /d	Laparotomy, cholecystectomy, debridement	Died at 2 M
36	Ho et al.	Stomach	Gastric Bx	None	NI	NR	IV AMB 0.5 g/kg/ d, 28 d	Colectomy, none on stomach	Alive
37	Vaiphei et al.	Stomach	Autopsy: stomach	None	NI	NR	None (post-motem diagnosis)	None	Dead, post-motem diagnosis
38	Chung et al.	Stomach, liver, spleen	Gastric, liver, spleen Bx	None	NI	HIV	IV AMB, 2 w	None	Alive
39	Shiva Prasad et al.	Stomach	Gastric Bx	None	NI	NR	IV AMB	None	Dead
40	Azhar et al.	Stomach	Gastric sample	None	NI	Peritoneal fluid: *E. cloacae*/ then *Enterobacter*, *E.faecalis*, Bile*: K. pneumoniae*/	IV AMB 1.5 mg/kg/d, 18 d then PO PSZ 200 mg x 4/d	Total gastrectomy, pancreas, anterior Abd wall, splenic necrosis debridement	Alive
41	Nandu et al.	Stomach	Gastric Bx	None	NI	NR	None (family refuse)	None	Dead, d 12 post diagnosis

42	Small et al.	Stomach	Gastric Bx	None	NI	NR	IV AMB	None	NR
43	Feng et al.	Stomach	Gastric Bx	None	NI	Nosocomial pneumonia *Staphylococcus aureus*, *Hemophillus influenzae*	IV L AMB 200 mg/d, 14d	None	Alive
44	Johnson et al.	Stomach	Gastric samples	None	NI	NR	PSZ	Total gastrotomy	Alive
45	Paydar et al.	Stomach	Gastric Bx and sample	None	NI	NR	IV LC AMB	Total gastrectomy	Alive
46	Pruthvi et al.	Stomach	Gastric Bx<br>No vascular invasion	None	NI	NR	None	None	Alive
47	Shenoi et al.	Stomach	Gastric Bx	Fungal PCR (method NS)/gastric brushing	*Rhizopus microsporus*	Peritonitis:<br>*K. pneumoniae*<br>Gastric: *C. glabrata*	IV L AMB 5 mg/kg/d, 6M +<br>MCF 3.5 mg/kg/d<br>PO PSZ (400 mg x2/d), 12 M	None (parents refuse)	Alive
48	Woo et al.	Stomach	Gastric Bx	Positive culture/ Bx<br>Identification:<br>ITS1-5.8S–ITS2 rDNA, partial EF1α<br>+ partial β-actin genes<br>PCR+sequencing	*Lichteimia (*formerly *Absidia) hongkongensis*	NR	IV AMB	None	Alive
49	Chhaya et al.	Stomach	Gastric Bx	Negative culture/gastric biopsy	NI	*Candida* like yeasts/gastric Bx<br>*E. coli* urosepsis	IV AMB, 4 w	None	Alive
50	Chang et al.	Stomach	Gastric Bx	Positive culture	NS	NR	IV L AMB	None	Alive
51	Dutta et al.	Stomach	Gastric Bx	None	NI	NR	IV AMB, 14 d	None	Alive
52	Muthuswamy et al.	Stomach	Gastric Bx	None	NI	Pneumonia, *Acinetobacter*, *Pseudomonas*	IV AMB	None	Dead
53	Ryan et al.	Stomach	Gastric, spleen samples	None	NI	HIV, HCV	IV AMB	None	Dead
54	Corey et al.	Stomach	Gastric Bx<br>No angioinvasion	Positive culture	*Rhizopus* sp.	Candidal esophagitis	IV AMB	None, because of end-stage liver disease	Dead

55	Irtan et al.	Stomach	Gastric sample	Positive culture<br>Identification:<br>rDNA ITS1-5.8S-ITS2 region PCR + sequencing	*Lichteimia (*formerly *Absidia) ramosa*	NR	IV L AMB 10 mg/kg/d, 2M<br>then 7.5 mg/kg/d, 2M<br>then 7.5 mg/kg/w, 2M	None	Alive
56	Katta et al.	Stomach	Gastric Bx	None	NI	NR	IV L AMB 5 mg/kg/d then 5 mg/kg x 3/w, 4 M<br>+ MCF 100mg/d 1 M<br>then PO PSZ, 200 mg x 4/d, 5M	None (patient refuse)	Alive
57	Machicado et al.	Stomach	Gastric Bx	None	NI	NR	IV L AMB, MCF	None	Alive
58	Bini et al.	Stomach	Gastric sample	None	NI	NR	IV L AMB	Total gastrectomy	Alive
59	Kaiser et al.	Stomach	Gastric sample	Negative pan-fungal PCR (target gene NS)/FFPET	NI	HBV	IV L AMB 7.5 mg/kg/d, 5 w<br>then PSZ 800 mg/d, 7 weeks	Ulcer resection	Alive
60	Lee et al.	Stomach	Gastric sample	None	NI	NR	IV L AMB, 5 mg/kg/day, 3 w	Subtotal gastrectomy	Alive
61	Kulkarni et al.	Stomach	Gastric Bx	None	NI	NR	None (post-mortem diagnosis)	Perforated gastric ulcer resection	Dead, post-mortem diagnosis
62	Nandwani et al.	Stomach	Gastric Bx	None	NI	CMV gastritis	IV L AMB 3 mg/kg/d	Distal gastrectomy	Dead
63	Nasta et al.	Stomach	Gastric Bx	None	NI	NR	None	Partial gastrectomy<br>Partial diaphragmatic excision	Dead 3 d after surgery<br>Post-mortem diagnosis
64	Raviraj et al.	Stomach	Gastric samples	None	NI	NR	IV AMB 50 mg/d, (total 3 g), 8 w then<br>PSZ, 2 w	Partial gastrectomy	Alive
65	Lin et al.	Stomach	Gastric Bx	None	NI	NR	None (patient refuse)	None	Alive
66	Mittal et al. R	Stomach	Gastric Bx	None	NI	NR	IV AMB	Distal gastrectomy<br>+ transverse colon segmental resection	Dead
67	Ravi et al.	Stomach	Gastric, samples	Positive culture of abdominal incision wound	*Rhizopus* sp.	Pleurisy:<br>*Streptococcus viridians* +<br>*C. glabrata*<br>Gastric: *C. glabrata*	None	None	Dead, 45 d after admission
68	Tathe et al.	Stomach	Gastric Bx<br>No vascular invasion	None	NI	NR	Yes (NS), 3 w	None	Alive

69	Chow et al.	Stomach	Gastric sample	None	NI	Enterococcus urinary infection	None	Sub-total gastrectomy	Died
70	Chugh et al.	Stomach	Liver Bx	None	NI	NR	PO PSZ, not taken	None	Alive
71	Galvan-Fernandez et al.	Stomach	Gastric post-mortem sample	None	NI	NR	None (post-mortem diagnosis)	None	Dead, post-mortem diagnosis
72	Grimaldi et al.	Stomach	Gastric Bx,<br>Stomach, spleen, peritoneal samples	None	NI	NR	IV L AMB + PSZ	Gastrectomy and splenectomy<br>Interferon-γ	Alive
73	Lyo et al.	Stomach	Gastric Bx	Sputum culture	*Rhizopus* sp.	Polymicrobial pneumonia	None	None	Alive
74	Metussin et al.	Stomach	Gastric Bx	None	NI	*S. aureus* bacteraemia	IV L AMB	None	Dead 2 w after admission
75	Nasa et al.	Stomach	Gastric Bx	None	NI	NR	IV AMB	None	Dead
76	Sanchez-Velasquez et al.	Stomach	Gastric sample	None	NI	NR	None	Total gastrectomy	Dead, 8 d after surgery
77	Suhaildeen et al.	Stomach	Gastric sample<br>(2 previous inconclusive Bx)	None	NI	NR	IV AMB	Total gastrectomy	NR
78	Abreu et al.	Stomach	Gastric Bx	None	NI	NR	IV AMB, total dose 2,8 g, 6 w	total gastrectomy	Alive
79	Alfano et al.	Stomach	Gastric Bx	Mucormycetes PCR/ gastric tissue	*Rhizopus* sp.	NR	IV L AMB 5 mg/kg/d, 5 d<br>then PO PSZ 100 mg x 3 /d	None	Alive
80	Chang et al.	Stomach	Gastric Bx	None	NI	NR	IV L AMB 5 mg/kg/d, 6 w	None	Alive
81	Kim C et al.	Stomach	Gastric Bx	None	NI	NR	None	Total gastrectomy<br>2 M after DG	Alive
82	Termos et al.	Stomach	Gastric sample	None	NI	NR	IV L AMB 5 mg/kg/d	Total gastrectomy	Dead, 21 d after surgery, sepsis
83	Adhikari et al.	Stomach	Gastric Bx<br>No vascular invasion	None	NI	*H. pylorii* gastritis	IV L AMB 5 mg/kg d1 then 10 mg/kg, 13 d<br>then PSZ 300 mg/d, 2 M	None	Alive
84	Gani et al.	Stomach	Gastric Bx	Positive culture	*Rhizopus* sp.	NR	IZV 372 mg /8 h, 48h then<br>372 mg /d lifelong	None	Alive

85	Guzman Rojas et al.	Stomach	Gastric Bx	Positive culture/gastric sample	*Rhizopus* sp.	HIV	IV AMB	Total gastrectomy	NR
86	Lankarani et al.	Stomach	Gastric Bx	None	NI	NR	IV L AMB, 8 w<br>then PO PSZ	None	Alive
87	Malek et al.	Stomach	Gastric sample	None	NI	Pulmonary *Mycobacterium tuberculosis* complex	IV L AMB 7.5 mg/kg/d<br>then PO PSZ (suspension) 200 mg/6 h per J-tube	Total gastrectomy	Alive
88	Peng et al.	Stomach	Gastric Bx	None	NI	NR	IV L AMB 2 mg/kg/d + PSZ 100 mg 3xd	None	Alive
89	Sharaan et al.	Stomach	Gastric sample	None	NI	NR	IV L AMB<br>then IZV	Total gastrectomy	Alive
90	Uchida et al.	Stomach	Gastric Bx	Positive culture<br>Identification:<br>ITS and D1/D2 rDNA regions PCR + sequencing	*Mucor indicus*	CMV	IV AMB 5 mg/kg/d	None	Alive (dead 159 d after diagnosis)
91	Monte Junior et al.	Stomach	Gastric Bx	None	NI	SARS Cov2	None (post-mortem diagnosis)	None	Dead (post-mortem diagnosis)
92	Hameed et al.	Stomach	Gastric Bx	None	NI	NR	None (post-mortem diagnosis)	Total gastrectomy	Dead (post-mortem diagnosis)
93	Jaju et al.	Stomach	Gastric, sample	Positive culture	*Mucor* sp.	NR	IV AMB 0.5mg/kg/day	Partial gastrectomy	Alive
94	Jung et al.	Stomach	Gastric Bx	None	NI	NR	IV L AMB (5 mg/kg/d), 8w	None	Alive
95	Naqvi et al.	Stomach	Gastric Bx	Positive culture and PCR biopsy specimens	*R. microsporus*	NR	IV AMB 5 mg/kg/d<br>switch to PSZ 300 mg/d<br>total duration 16w	None	Alive
96	Rivas et al.	Stomach	Gastric Bx	None	NI	NR, septic shock	None	None	Dead
97	Sharma et al.	Stomach	Gastric sample	None	NI	NR	IV L AMB 5 mg/kg/d, 6 w	Total gastrectomy	Alive
98	Sharma et al.	Stomach	Gastric sample	None	NI	NR	IV L AMB 5 mg/kg/d, 6 w	Total gastrectomy	Alive

99	Ghuman et al.	Stomach	Gastric sample	None	NI	NR	None	Gastrectomy, then perforated ileal resection	Dead
100	Huang et al.	Stomach	Gastric Bx	None	NI	Not specified bacterial infection	None	Subtotal gastrectomy	Alive
101	Huang et al.	Stomach	Gastric sample	None	NI	NR	IV AMB, 1M	Total gastrectomy, jejunostomy	Alive
102	Rai et al.	Stomach	Gastric Bx	None	NI	NR	FCZ	Gastric perforation repair	Alive
103	Yuvaraj et al.	Stomach	Gastric Bx	None	NI	NR	IV AMB 300 mg, 1 d	Planed gastrectomy + splenectomy	Dead before surgery
104	Bhaskar et al.	Stomach	Gastric sample	None	NI	NR	IV AMB (7 d prior to surgery); then PSZ oral suspension	Subtotal gastrectomy	Dead
105	Chauhan et al.	Stomach	Gastric Bx	None	NI	SARS Cov2	IV L AMB, 28d then PO PSZ	None	Alive
106	Danion et al.	Stomach	Gastric Bx	PCR and sequencing ITS rDNA/ FFPET *Positive Mucorales* PCR/blood	*R. microsporus*	COVID-19 Associated Pulmonary Aspergillosis	IV L AMB 5 mg/Kg/d	None (peri-mortem diagnosis)	Dead 12h after the ATFT start
107	Khsiba et al.	Stomach	Gastric Bx	Positive DE+culture	*Mucorales*	NR	IV AMB 5 mg/day 4 days than 25 mg/day (0.4 mg/kg/day)	Total gastrectomy, 2 M after DG	Dead after surgery
108	Khsiba et al.	Stomach	Gastric Bx	None	NI	NR	None	None	Dead d 8 after admission (hemophagocytic syndrome)
109	Sachan et al.	Stomach	Gastric Bx	None	NI	NR	IV L AMB,	None	NR
110	Sachan et al.	Stomach	Gastric Bx	None	NI	NR	IV L AMB, 4 w then PSZ	None	NR
111	Safwan et al.	Stomach	Gastric Bx	None	NI	Cutaneous, *K. pneumoniae*	None (post-mortem diagnosis)	None	Dead, Post-mortem diagnosis
112	Albtoosh et al.	Stomach	Gastric Bx	None	NI	NR	IV L AMB 5mg/kg/day, 4 w	None	NR, alive at 1 M
113	Arora et al.	Stomach	Gastric sample	None	NI	*K. pneumoniae* septicaemia	IV L AMB	Proximal gastrectomy	Dead
114	Bhowmik et al.	Stomach	Autopsy: stomach	None	NI	NR	NR	None (post-mortem diagnosis)	Dead at d 8, post-mortem diagnosis

115	Khanna et al.	Stomach	Gastric Bx	None	NI	NR	IV AMB	None	Alive
116	Kim J H et al.	Stomach	Gastric Bx, then sample	None	NI	NR	IV L AMB	Total gastrectomy D14 of AFT	NR
117	Malakar et al.	Stomach	Gastric Bx	None	NI	NR	IV AMB, 13 d then PSZ 3 M	None	Alive
118	Marco et al.	Stomach	Gastric Bx, sample	Positive culture / Bx, sample	*R. microsporus*	NR	IV L AMB 10 mg/kg/d, 6 w +IZV then PSZ, 6 w	Gastrectomy	Alive
119	Chaudhari et al.	Stomach	Gastric Bx	None	NI	NR	NR	NR	Dead
120	de Lucena et al.	Stomach	Gastric Bx	None	NI	NR	IV AMB, 30 d then IZV, 2 M	None	Alive
121	He et al.	Stomach	Gastric Bx	None	NI	SARS Cov2	IV L AMB	None	Alive
122	Huang et al.	Stomach	Gastric Bx	None	NI	NR	IV AMB	None	Alive
123	Ji R.	Stomach	Gastric sample	None	NI	NR	PSZ (100 mg x 2/d) instillation in feeding tube	Gastrectomy (d5 post endoscopy)	Dead, MOF
124	Lalwani et al.	Stomach	Gastric sample	None	NI	NR	IV L AMB	Distal gastrectomy, transverse colectomy	Dead 2 d after surgery Post-mortem diagnosis
125	Pérez Fernández et al.	Stomach	Gastric Bx	None	NI	NR	NR	NR	NR
126	Raju et al.	Stomach	Gastric Bx	Positive culture/Bx Identification: D1/D2 region of the LSU rDNA PCR+sequencing	*Syncephalastrum racemosum*	NR	IV AMB 1 mg/kg/d	ubtotal gastrectomy	Alive
127	Knoop et al.	Stomach, lung (probable)	Gastric Bx	Positive culture	*R. microspores* var. *rhizopodiformis*	Bronchitis, *P. aeruginosa*	IV LC AMB, 3 mg/kg/d, 6w	Partial gastrectomy D7 of AFT	Alive
128	Taams et al.	Intestine (ileum)	Ileum sample	Culture result NR	NI	Polymicrobial peritonitis	None (post-mortem diagnosis)	Ileal perforation repair, necrosis resection	Dead, post-mortem diagnosis
129	Sharma et al.	Intestine	Intestine sample	None	NI	NR	None	Necrotized intestine resection	Dead
130	Lalwani et al.	Intestine	Jejunal sample	None	NI	NR	IV L AMB (7 mg/kg/d), 4 w	Resection of perforation of a segment of the Roux-loop	Alive

131	Lalwani et al.	Intestine	Cecum sample	None	NI	NR	IV AMB	Right hemicolectomy	Alive
132	Saltmarsh et al.	Intestine	Bowel Bx	None	NI	HIV	IV AMB	Proximal jejunum perforations debridement then small bowel resection, sub-total gastrectomy	Dead, 5 d after AFT start
133	Ju et al.	Intestine (colon)	Colon Bx	None	NI	Digestive CMV infection	IV AMB 1mg/Kg/d	None, Transplant nephrectomy for Transplant dysfunction	Alive
134	Kaneko et al.	Intestine (colon)	Colon samples	Positive culture/drainage from Douglas' pouch Identification: ITS and D1/D2 rDNA regions + elongation factor 1α of *Lichteimia* PCR + sequencing + β-tubulin of *Aspergillus*	*L. ramosa*	*A. calidoustus*	IV L AMB 8 mg/kg/d, 72 d	Subtotal colectomy cecum to upper rectum	Alive
135	Nidhi et al.	Intestine (colon)	Colic sample	None Negative PCR (method NS)/ FFPET	NI	NR	None (post mortem diagnosis)	Colonic mass resection	Dead, post-mortem diagnosis
136	Hyvernat et al.	Intestine (colon)	Colon sample	Positive DE + culture Identification: ITS rDNA region PCR and sequencing	*R. microsporus*	NR	None (perimortem diagnosis)	Colectomy	Dead, perimortem diagnosis
137	Wotiye et al.	Intestine (colon)	Intestine Bx	None	NI	NR	IV L AMB 5 mg/kg/d, total dose 3.2 g	None	Alive
138	Liu et al.	Intestine (colon)	Colic Bx	Positive culture/ colic Bx Identification: 18S rRNA region PCR and sequencing	*L. ramosa*	NR	PSZ, 3 d	None	Dead, MOF
139	Mungazi et al.	Intestine (duodenum)	Duodenum Bx	None	NI	NR	None (post mortem diagnosis)	None	Dead, post-mortem diagnosis
140	Sun et al.	Intestine (duodenum, jejunum)	Duodenal sample	None	NI	Abd drainage fluid: *C. albicans*, *K. pneumoniae*, *A. baumannii*	IV AMB 5mg/d increased to 20mg/d, 20 d +PO mycostatin 4 w	Duodenal lesions +proximal jejunum resection	Dead

141	Poyuran et al.	intestine (Ileo-caecal)	Ileal sampleimage not typical	None	NI	NR	Yes, NS	Right hemicolectomy	Dead
142	Bhat et al.	Intestine (ileo-colic)	Ileum, colon samples	None	NI	NR	IV AMB, 1 M	Terminal ileum resection, limited right hemicolectomy	Alive
143	Taams et al.	Intestine (ileum)	Ileum sample	Culture result NR	NI	Polymicrobial peritonitis	None (post-mortem diagnosis)	Ileal perforation repair, necrosis resection	Dead, post-mortem diagnosis
144	Gupta et al.	Intestine (Ileum)	Ileal sample	None	NI	*E. coli* peritonitis	IV AMB 1 mg/Kg/d, 6 d	Ileal resection	Lost of follow up
145	Aruni et al.	Intestine (ileum)	Ileal sample	None	NI	NR	None	Perforated ileal segment resection	Alive
146	Himaal et al.	Intestine (ileum)	Ileal sample	None	NI	H1N1 *influenzae*	IV AMB, 3 w<br>then PO PSZ	Ileal resection	Alive
147	Manda et al.	Intestine (ileum)	Ileum Bx	NR	NI	NR	NR	NR	NR
148	Zulpi et al.	Intestine (ileum)	Ileum sample	None	NI	NR	IV AMB, 4 w	Proximal ileum partial resection	NR, alive at 1 M
149	Chethan et al.	Intestine (ileum, cecum)	Ileum + cecum Bx	None	NI	NR	IV AMB 1,5 mg/kg/d	None	NR, alive at d 14 post ATFT start
150	Yadav et al.	Intestine (ileum, colon)	Ileum, colon samples	None	NI	NR	IV L AMB 200 mg/d	Ileotomy, tranverse colectomy	Dead
151	Sarkadeh et al.	Intestine (ileum, colon)	Ileum sample	None	NI	NR	NR	Terminal ileum + ascending colon resection	Dead
152	Martinello et al.	Intestine (jejunum)	Jejunum sample	Identification: PCR and sequencing ITS rDNA region/ (FFPET)<br>Negative culture, jejunum sample	*R. arrhizus (*formerly *oryzae)*	*C. albicans*	IV L AMB 5 mg/kg/d, 5w<br>then PO PSZ 200 mg x 4/d, 25 w	Jejunum resection	Alive
153	Kumar et al.	Intestine (jejunum)	Jejunum sample	None	NI	NR	IV AMB	Jejunum resection	Alive
154	Budhiraja et al.	Intestine (jejunum)	Jejunum sample<br>No vascular invasion	None	NI	NR	IV AMB	Two jejunal strictures (20 cm) resection	Alive
155	Bhana et al.	Intestine (jejunum)	Jejunum sample	None	NI	NR	IV AMB, 21 d	Abnormal bowel resection	NR, alive at 1M
156	Watson et al.	Intestine (jejunum)	Jejunum sample<br>gastric +peristomal Bx	Positive sample and cultures /Bx	*Mucor* sp.	NR	IV L AMB, 8 w<br>PO PSZ, 10 M	Bowel resection	Alive

157	Paliwal et al.	Intestine (jejunum, ileum)	Bowel sample	None	NI	SARS Cov2	None (post-mortem diagnosis)	Jejunum and ileum repair, pyoperitoneum evacuation, gangrenous bowel resection	Dead, 1 d after surgery, post-mortem diagnosis
158	Horowitz et al.	Intestine (sigmoid colon)	Autopsy: Intestine (sigmoid colon)	None	NI	NR	None (post-mortem DG)	None	Dead
159	Sriperumbuduri et al.	Intestine (sigmoid colon)	Sigmoid, colon Bx	None	NI	NR	IV AMB 1mg/kg/d, total dose 2.8 g	None	Alive
160	Singh et al.	Intestine (sigmoid colon)	Sigmoid sample	None	NI	SARS Cov2	IV L AMB 3 mg/kg increased to 5 mg/kg, 7 d + PSZ 400 mg x 2/d, 14 d	Sigmoid colectomy	Alive
161	Gupta et al.	Intestine (small intestine, colon)	Colic sample	None	NI	*E. coli* peritonitis	IV L AMB 5 mg/kg/d, 2d	Colectomy, none on stomach	Dead
162	Sharma et al.	Intestine, cecum	Intestine, cecum samples	None	NI	NR	None	Ileal lump, caecum resection	Dead
163	Nagy-Agren et al.	Small + large intestine	Autopsy	None	NI	HIV	None (post mortem diagnosis)	None	Dead, post-mortem diagnosis
164	Cebisli et al.	Small + large intestine	Small + large intestine Bx	None	NI	NR	IV AMB, 6 w	Small and large bowel necrosis rejection	Dead
165	Saraf et al.	Small + large intestine	Bowel samples	None	NI	NR	None	Terminal ileum, caecum, part of ascending colon resection	Dead
166	Eiser et al.	Small intestine	Small intestine sample	None	NI	NR	None	Small intestine resection	NR
167	Yinadsawaphan et al.	Small intestine	Small intestine sample	Positive peritoneal fluid culture ITS1–5.8S-ITS2 r DNA region PCR+sequencing	*R. microsporus*	HIV	IV L AMB 300 mg/ d (5 mg/kg/day), 12 d	Segmental resection of the small bowel then total small bowel resection	Dead
168	Li et al.	Oesophagus	Oesophagus Bx Image not typical, vacuolized fungal structures	Negative culture / oesophagus Bx,	NI	NR	IV AMB, 1 w then PSZ, 3 w	None	Alive

169	Evert et al.	Oesophagus	Autopsy: oesophagus	Identification: PCR and sequencing ITS rDNA/ oesophagus FFT or FFPET	*Mucor* sp.	*A. fumigatus*	None	None	Dead, septic MOF, post-mortem diagnosis
170	Danion et al.	Digestive, site NS	Digestive (site NS)	Molecular identification *Rhizopus* specific PCR+sequencing	*R. microsporus*	NR	IZV	NS (pooled data)	Alive
171	Marcó del Pont et al.	Liver	Liver (2nd transplant) sample	Wound DE, culture	*Mucor* sp.	*Serratia marcescens*/wound	None	Liver de-transplantation	Dead, post-mortem diagnosis
172	Mekeel et al.	Liver	Liver sample	Positive culture	*Mucor* sp.	NR	NR	Hepatic lobectomy	Dead (10 d after surgery)
173	Zhan et al.	Liver	Liver sample (post-mortem)	Negative culture	NI	Liver and kidney aspergillosis	None	Transplant nephrectomy	Dead
174	Abboud et al.	Liver	Liver Bx	None	NI	NR	IV L AMB 5mg/kg/d, 60d +local/drain L AMB and polymixin B then PO PSZ 200 mgx4/d, 6M	None	Alive
175	Chowdhary et al.	Liver	Liver Bx	None	NI	NR	IV L AMB 5 mg/kg/d + PO PSZ 200 mg x 2/d (5ml), 3 M then PSZ 200 mg x 2/d, 3 M	None	Alive
176	Teira et al.	Spleen	Spleen sample	None	NI	HIV, Oral candidiasis, cerebral toxoplasmosis	IV AMB total dose 650 mg	Splenectomy	Alive
177	Chakrabarti et al.	Spleen	Spleen, flank sample	Pus DE + culture ITS rDNA region PCR and sequencing	*Apophysomyces elegans*	NR	IV AMB, 0,5 mg/kg/d	Abscess drainage, local debridement	Dead, post-mortem diagnosis
178	Gupta et al.	Spleen	Peri splenic collection smear, splenic sample	None	NI	NR	IV AMB 50 mg/day, total dose of 3g, 60 days	Splenectomy, left nephrectomy	Alive
179	O'Connor et al.	Spleen	Splenic abscess	Negative culture/gastric, duodenal, splenic samples	NI	Peritoneal: *Aspergillus* positive PCR	IV L AMB 5 mg/kg/d	Gastrostomy, duodenostomy, splenectomy	Alive
180	Luo et al.	Spleen	Splenic sample	None	NI	NR	IV L AMB + PSZ then PO PSZ	Splenectomy	Alive
181	Jain et al.	Mesenteron	Mesenteric sample	None	NI	SARS Cov2	None (post mortem diagnosis)	Right hemicolectomy, partial resection of distal ileum	Dead, post-mortem diagnosis

182	Okhuysen et al.	Intra-abdominal	Myonecrosis sample	Positive DE+culture, myonecrosis sample	*A. elegans*	*Enterobacter sp*., *Enterococcus* sp. Myonecrosis	IV L AMB, 5 mg/kg/d, 8 w AMB irrigation/nephrostomy tube	Extended surgical debridement, interferon ɣ, hyperbaric oxygen	Alive
183	Durila et al.	Intra-abdominal	NR	Abdominal drainage fluid, abdominal wound cultures	*R. microsporus*	VHA	Yes (NS) 2,5 M	NR	Alive
184	Khanna et al.	Peritoneal	NR	Positive peritoneal fluid culture	*Mucor* sp.	NR	None	Catheter removal	Alive
185	Polo et al.	Peritoneal	NR	Negative routine cultures Positive culture /peritoneal concentrated fluid, catheter cuff	*Rhizopus* sp.	NR	IV AMB	Peritoneal catheter removed	Dead (intestinal perforation)
186	Fergie et al.	Peritoneal	NR	Positive culture /peritoneal fluid, then catheter tip	*Mucor* sp.	NR	IV AMB 0,25 then 0,5 mg/kg/d	Tenckhoff catheter removal	Dead
187	Adam et al.	Peritoneal	NR	Positive culture /peritoneal fluid	*Mucor ramosissimus*	NR	IV AMB	none	Alive
188	Khan et al.	Peritoneal	NR	Positive peritoneal fluid culture	*L. corymbifera*	NR	NR	NR	NR
189	Monecke et al.	Peritoneal	NR	Positive culture/Abd gangrenous area swab Identification: ITS rDNA region pan-fungal PCR and sequencing + hybridization against a set of specific probes on a DNA microarray	*R. microsporus*	NR	IV AMB 10 mg d 1, then 50 mg/d AMB + Lavasept peritoneum irrigation (treatment at d 41 POT)	Transplant nephrectomy, d 14 POT	Dead 45 d after KT, MOF
190	Pimentel et al.	Peritoneal	NR	Negative DE+positive enrichment (BACTEC bottles) culture/peritoneal fluid	*Cunninghamella bertholletiae*	NR	VRZ 200 mg x 2/d, 90 d	Tenckhoff catheter removed Peritoneal fluid drainage	Alive
191	Nayak et al.	Peritoneal	Peritoneum Bx, no fungi evidence	Positive PD fluid sediment DE + culture	*Rhizopus* sp.	NR	IV L AMB total dose 1 g, 4 w	Tenckhoff catheter removed	Alive
192	Bhutada et al.	Peritoneal	NR	Positive DE + culture/peritoneal fluid	*C. bertholletiae*	NR	Intraperitoneal FCZ 200 mg/d, 8 d then ITZ 100 mg x 2/d, 8 d	None, Tenckhoff catheter removed	Alive

193	Pamidimukkala et al.	Peritoneal	NR	Positive DE+culture, CAPD catheter tip *Apopysomyces* ITS rDNAregion PCR+sequencing	*A. variabilis*	NR	IV AMB	None	Dead
194	Dalgic et al.	Peritoneal, liver (possible)	NR	Positive culture/peritoneal fluid	*R. arrhizus (formerly oryzae)*	NR	IV AMB 5 mg/kg/day, 8 w	Ascites drainage	Alive, liver lesions resolving
195	Adam et al.	Abd wall (skin, muscle)	Abd wall sample	Positive culture/Abd sample	NS	NR	IV AMB	Tissue resection	Alive
196	Verma et al.	Abd wall	Abd wall sample	None	NI	NR	IV AMB	Debridement	Dead
197	Chakrabarti et al.	Abd wall	Skin, subcutaneous necrotic samples	Positive culture/Abd wall samples	*Saksanea vasiformis*	NR	NR	Extensive debridements	Dead, postoperative hours
198	Mathews et al.	Abd wall	Abd wall necrotic tissue	Positive DE+culture/Abd wall necrosis	*A. elegans*	P. aeruginosa, E. fecalis	IV AMB, total dose 2g	Extensive debridements	Alive
199	Kumar et al.	Abd wall	NR	Positive DE+culture/Abd wall samples	*Rhizopus* sp.	NR	IV AMB 0.25 to 1 mg/kg/d, total dose 1 g then IV L AMB few days	Extensive debridments	Alive
200	Thami et al.	Abd wall	Abd wall sample	Positive DE+culture/ Abd wall necrotic samples	*L. corymbifera*	NR	Topical AMB + PO aqueous solution of potassium iodide (1 mg ⁄ mL), 4 w	Extensive debridments	Alive
201	Kerbaul et al.	Abd wall	Abd wall Bx	None	NI	NR	IV L AMB 250 mg/d	Necrotic wound excision	Dead
202	Padmaja et al.	Abd wall	Necrotic tissue	Positive DE+culture/ Abd wall necrotic samples	*S. vasiformis*	NR	IV AMB	Extensive debridments	Dead, patient left against advice
203	Belfiori et al.	Abd wall	NR	Positive culture/Abd wall abscess fluid	*L. corymbifera*	*E. faecium, P. aeroginosa, C. albicans*	IV L AMB 5 mg/Kg/d, 40 d	Abdominal wall drainage	Alive
204	Tilak et al.	Abd wall	Necrotic tissue	Positive DE+culture/ Abd wall necrotic samples	*R. arrhizus (formerly oryzae)*	NR	IV AMB, 6d	Extensive debridments	Dead
205	Tilak et al.	Abd wall	Necrotic tissue	Positive DE+culture/ Abd wall necrotic samples	*R. arrhizus (formerly oryzae)*	NR	IV L AMB 4 mg/kg/d, 14 d	Extensive debridments	NR, favorable
206	Tapish et al.	Abd wall	Abd wall necrotic tissue	Positive DE+culture/ Abd wall necrotic samples	*Mucorales*	NR	IV L AMB, total dose 1.5 g	Extensive debridments	Alive

207	Alharbi et al.	Abd wall	Abd wall sample	Positive DE +culture/ Catheter cuff, Abd wall	*Rhizopus* sp.	NR	IV AMB 1mg/kg/d then 2mg/kg/d, 33 d<br>IV PSZ 300 mg every 12 h, 21 d then<br>PO PSZ 400 mg x 2/d, 16 d; then 300 mg x 2/d, 109 d	Debridement of necrotic tissue to Abd wall fascia	Alive
208	Pamidimukkala et al.	Abd wall	NS (pooled data)	Positive DE+culture / fasciitis sample	*A. elegans*	NR	IV AMB	Surgical debridement	Dead
209	Pamidimukkala et al.	Abd wall (fasciitis)	NS (pooled data)	Positive DE+culture/ fasciitis sample	*A. elegans*	NR	IV AMB	Surgical debridement	Dead
210	Pamidimukkala et al.	Abd wall (fasciitis)	NS (pooled data)	Positive DE+culture/ fasciitis sample	*A. elegans*	NR	IV AMB	Surgical debridement	Dead
211	Pamidimukkala et al.	Abd wall (fasciitis) xiphisternum to the suprapubic region, between flanks	NS (pooled data)	Positive DE+culture/ fasciitis sample	*A. elegans*	NR	IV AMB	Surgical debridement	Lost of follow up (left against medical advice)
212	Pamidimukkala et al.	Abd wall, omentum, intestine, kidney, chest wall	NS (pooled data)	Positive DE+culture, tissue samples	*A. elegans*	NR	IV AMB	Debridement of the chest wall, nephrectomy (left), resection of the gangrenous colon and omentum	Lost of follow up (left against medical advice)
213	Taams et al.	Abd wall, intestine (ileum, colon)	Ileum, colon, abdominal wall samples	None	NI	NR	None (post-mortem DG)	Ileal repair, large right paracolic abscess drainage, abdominal wall necrosis resection	NR
214	Taams et al.	Abd wall, intestine (ileum, colon)	Ileum, colon, abdominal wall samples	Negative fungal culture	NI	Multiples bacteria	IV AMB	Colic and kidney lacerations repair, perirenal area drainage, abdominal wall necrosis resection	Dead
215	Ram et al.	Abd wall, peritoneal	Wound tissue	Positive culture/catheter tip	*Mucorales*	NR	AMB wound treatment	PD catheter removal<br>Wound cleaning + dressing, 3 w then surgical debridement	Lost of follow up

216	Patel et al.	Abd wall, intestine (colon)	Necrotic Bx + tissue, colon	None	NI	Surgical site scar*, E. coli*	IV AMB 1mg/kg/d	Extensive debridements + right hemicolectomy	Dead d 8 after last surgery
217	Zhao et al.	Abd wall, kidney	Abd wall necrotic sample then kidney Bx	Positive culture/Abd wall necrosis	Mucorales	NR	IV L AMB 25 mg/d then 50 mg/d	Debriedments, then transplant nephrectomy	Alive
218	Paonam et al.	Abd wall, kidneys	Kidneys, abdominal wall samples	Negative fungal cultures	NI	NR	IV AMB	Surgical drainage then necrotic material excision	Dead, post-mortem diagnosis
219	Narayanaswamy et al.	Abd wall, intestine (colon), spleen, kidney, retroperitoneal (probable)	Kidney sample	None	NI	Retroperitoneal *E. coli* infection	IV AMB, 3w	Nephrectomy, hemicolectomy, splenectomy	Alive
220	Stein et al.	Stomach, peritoneal	Autopsy	None	NI	NR	NR	NR	Dead
221	Lawson et al.	Oesophagus, stomach, intestine (colon)	Autopsy: oesophagus, stomach, colon	None	NI	NR	None	None	Dead, post-mortem diagnosis
222	Lawson et al.	Stomach, (intestine) colon	Autopsy: stomach, colon	None	NI	NR	None	None	Dead, post-mortem diagnosis
223	Eiser et al.	Liver, intestine (jejeunum, ileum)	Autopsy: liver, intestine (jejeunum, ileum)	None	NI	NR	None	None	Dead, post-mortem diagnosis
224	Branton et al.	IA abscess, Ileal wall	Autopsy: IA abscess, ileal wall samples	Positive peritoneal fluid culture	Rhizopus sp.	NR	Intraperitoneal AMB and IV AMB, total dose 500mg	None, Tenckhoff catheter removed	Dead (myocadiac infarct)
225	Nordén et al.	Intra-abdominal, pelvic, thoracic	IA tissue	Wound culture	*R. microsporus* var. *rhizopodiformis*		IV AMB 0.15-0.30 mg/kg/d, 3w	Extended surgical revision	Alive
226	Taams et al.	Intestine (colon), omentum	Colon, omentum samples	None	NI	***E. coli***, *B.fragillis* intra-abdominal	IV AMB	Colic necrosis, splenic flexure, necrotic omentum resection	Dead (penumonia)
227	Vadeboncoeur et al.	Intestine (colon) then intra-abdominal abscess	Colic then abdominal abscess samples	None	NI	NR	IV L AMB high dose	Subtotal colectomy, small bowel necrotic areas resection then abscess drainage	Alive

228	Hosseini et al.	Stomach, small and large intestine	bowel samples then gastric samples	None	NI	NR	IV AMB total dose 1 g	Subtotal colectomy then duodeno-gastrectomy	Dead
229	Guardia et al.	Kidney, peripancreatic tissue	Kidney Bx, then autopsy	None	NI	HIV	IV AMB	None	Dead
230	Herbrecht et al.	Oesophagus, stomach, mediastinum	NR	Positive culture/oesophagus, stomach, mediastinum	*Rhizopus* sp.	NR	IV AMB, total dose 240 mg then AMB Colloidal Dispersion 6,2 mg/kg, total dose 3,3 mg, 7 d	Mediastinum debridement	Dead
231	Nannini et al.	Peritoneal, intra-abdominal abscess	IA abscesses sample	Positive peritoneal fluid culture	*Mucor* sp.	NR	IV L AMB 5 mg/kg x 3 / w, 3 M	Tenckhoff catheter removal then pelvic collection surgical drainage (w 6 of AFT)	Alive
232	Serna et al.	Peritoneal, IA abscess	Pelvic collection	Positive peritoneal fluid culture	*Mucorales*	*E. coli* peritoneal infection	IV AMB then IV L AMB 5 mg/kg/d, 6w	PD catheter removal then transplant nephrectomy then abscesses surgical excision	Alive
233	Alkhunaizi et al.	Kidney, liver	KT sample, Liver Bx	None	NI	NR	IV AMB	Transplant nephrectomy	Alive
234	Sethi et al.	Liver, kidney	Kidney, liver samples	Tissue culture	*Rhizopus* sp.	NR	IV L AMB total dose 5 g then PSZ 300 mg /d, 6 M	Right nephrectomy, liver debridement	Alive
235	Deja et al.	Stomach, intestine (caecum, colon)	Gastric, intestine (caecum, colon) Bx	Positive Immunohistology for *Rhizomucor sp.* Positive culture Identification:ITS4-ITS5 PCR + sequencing	*M. indicus*	NR	IV L AMB 7.5 mg/kg /d, 33 d local AMB via the gastric tube (2 x 100 mg/d) +Granulocyte-macrophage colony-stimulating factor (GM-CSF)	Hemicolectomy	Alive
236	Manchikalapati et al.	Oesophagus, stomach, intestine (duodenum, ileum, cecum)	Gastroesophageal, gastric, duodenal Bx Oesophagus, stomach, ileum, caecam, peri renal samples	Positive culture/gastric mass	*Rhizopus* sp.	*Pseudomonas* sepsis	IV AMB	Total gastrectomy, right hemicolectomy, abscesses detersion	Alive, (dead, 106 d after surgery)
237	Jung et al.	Stomach, liver, Abd muscle layer and serosa	Gastric sample	None	NI	Peritonitis: *K. pneumoniae*, *S. aureus*, *P. aeruginosa*	IV AMB, 1M	Total gastrectomy	Dead 21 d, after admission

238	Sedlacek et al.	Peritoneal, IA abscess	Surgical abscess drainage	Positive DE+culture/ peritoneal fluid	*Mucor* sp.	NR	IV L AMB 10 mg/kg/d, 4 w then 5mg/kg/d, 8w<br>then PSZ 400 mg x2/d, 6 M	Tenckhoff catheter removal<br>then pelvic abscess percutaneous drainage then IA surgical drainage	Alive
239	Berne et al.	Stomach, Abd wall	Gastric, Abd wall samples	Positive Blood culture	*Mucor* sp.	Respiratory: *Seratia*, *Citrobacter*	IV AMB	Gastrectomy, partial oesophagectomy, pancreatectomy	Dead
240	Islam et al.	Stomach, spleen	Gastric, spleen samples	None	NI	NR	None (post-mortem diagnosis)	Total gastrectomy and splenectomy	Dead, post-mortem diagnosis
241	Mezhir et al.	Stomach, liver (d 35 after stomach)	Gastric Bx	Positive culture/ peritoneal fluid<br>Positive DE+ negative culture/Liver Bx	*Mucor* sp.	NR	IV L AMB 5mg/kg/d + PO AMB 50 mg/d<br>then IV L AMB 5mg/kg/d +PO PSZ 200mg x 4/d<br>then PO PSZ 400mg x 2/d, 1 Y	None (patient refuse)	Alive
242	Van Sickels et al.	IA, liver	Liver sample	Positive IA wound drainage DE+culture	*R. microspores* var. *rhizopodiformis*	NR	IV L AMB, high-dose + IV MCF standard dose; 8 wthen PO PSZ, 3 M	Liver, greater omentum debridement	Alive
243	Rudler et al.	Stomach, intestine (colic), spleen	Gastric, splenic, colic samples	PCR (method NS)/Gastric sample	*R. microsporus*	Vancomycin resistant *Enterococcus* (VRE) wound infection, staphylococcal bacteraemia	IV AMB	Gastrectomy, splenectomy then colectomy	Dead 1 d after surgery<br>Post-mortem diagnosis
244	Tan et al.	IA abscess, peritoneal	Pus fluid, peritoneal tissue sample	None	NI	NR	FCZ	Bowel resection, pus drainage	Dead
245	Enani et al.	Stomach, spleen	Gastric and spleen samples	None	NI	NR	None (post-mortem diagnosis)	Ulcer resection and splenectomy	Dead, post-mortem diagnosis
246	Gaut et al.	Retro-peritoneal, small and large intestine, stomach, liver, pancreas, peripancreatic fat, bladder, and femoral artery and vein	Retroperitoneal, small and large intestine tissue samples then<br>Autopsy: liver, pancreas, peripancreatic fat, stomach, bladder, small and large bowel, and femoral artery and vein	Positive culture/retroperitoneal sample	*R. microsporus*	VRE bacteraemia	IV L AMB 7.5 mg/kg/ d + Cas 70 mg d1 then 50 mg/d<br>IV PSZ 300 mg x 2/d then 300 mg/d, 2 d<br>switched to IV IZV 372 mg/8 h<br>Continuous intraperitoneal irrigation with L AMB 12.5 mg in 500 mL	Right hemicolectomy, segmental jejunal resection<br>Retroperitoneal debridement, ileal resection, right iliacus and psoas muscles partial resection,	Alive
247	Gupta et al.	Oesophagus, stomach, intestine (colon)	Oesophagus, stomach, intestine (colon) post-mortem samples	None	NI	NR	None (post-mortem diagnosis)	None	Dead, post-mortem diagnosis

248	Izaguirre-Anariba et al.	Stomach, bladder	Gastric, bladder Bx			NR	IV L AMB 5 mg/kg/d, 2 d<br>oral PSZ 400 mg x2/d, 6 d<br>bladder local AMB 200 ml, 5 d<br>IV IZV 372 mg /8h, 48 h<br>then to an PO of 372 mg/d, 6M	None	Alive
249	Aggarwal et al.	Intestine (colon)<br>IA abscess	Colic Bx<br>IA abscess	None	NI	Pneumonia + pleurisy:<br>*K. pneumoniae*	IV L AMB	Sigmoid hemicolectomy	Dead
250	Reis et al.	Intestine (ileum, colon), liver, lymph nodes	Ileum, colon, liver, lymph nodes samples	None	NI	NR	None (post-mortem diagnosis)	Yes, NS	Dead, post-mortem diagnosis
251	Rotundo et al.	Stomach, Abd cavity	Gastric Bx<br>Autopsy: Stomach, Abd cavity	None	NI	*Klebsiella* pneumonia	IV AMB then IZV	Total gastrectomy	Dead
252	Sehmbey et al.	Stomach, small intestine anastomosis, rectal stump	Gastric + bowel Bx	None	NI	NR	IV L AMB	Necrotic bowel resection	Dead
253	Shankaralingappa et al.	Intestine (ileum, colon), mesenteron	Ileum, colon, mesenteric vasculature samples<br>Previous (16 d ago)<br>Negative colon Bx	None	NI	NR	None	Ileum, transverse colon resection	Dead
254	Pamidimukkala et al.	Perineum, intestine (rectum, anal canal)	NS (pooled data)	Positive DE+culture, fasciitis sample	*A. elegans*	NR	IV AMB	Surgical debridement	Dead
255	Petrochko et al.	Intestine (sigmoid colon), Abd subcutaneous tissue	Colic Bx then colic, Abd samples	None	NI		IV L AMB 400mg /d<br>+ MCF 100mg /d	Left hemicolectomy after 27 d of AFT	Dead
256	Shah et aL	Intestine (colon), IA and pelvic abscess	Colic Bx than sample	Positive culture/ c olic sample then IA+pelvic abscesses	*Lichteimia* (formerly Absidia) sp.	NR	IV L AMB 500 mg/d, then IZV, then PSZ<br>then (after abscess diagnosis)<br>IV L AMB	Total colectomy	Alive
257	Chiang et al.	Intestine, (colon), IA abscess	Colon Bx, AI abscess	None	NI	NR	PO PSZ, 2 M	None	Alive
258	Hammami et al.	Stomach, spleen	Gastric, splenic samples	None	NI	NR	IV AMB 1 mg/kg/day, 70 d	Splenectomy and total gastrectomy	Alive
259	Martin-Blais et al.	Intestine (ileum, colon), kidney	Ileum, colon, kidney Bx	DG+identification:<br>NGS/ blood<br>PCR + sequencing ITS rDNA region/<br>kidney sample	*R. arrhizus (formerly oryzae)*	*S. maltophila*, *E. coli*	IV L AMB +ISV + CSF, 2M<br>then IV L AMB + PO ISV, 5 M	Nephrectomy	Alive

260	Varshney et al.	Kidney, mesenteron	Kidney, bowel mesentery samples	None	NI	SARS Cov2	None	Nephrectomy thenBowel gangrenous segment duodenum D3 to proximal jejunum (100 cm) resection	Dead
261	Danion et al.	Intestine (colon), peritoneal	Colic sample	Positive DE+culture/ peritoneal fluid<br>Mucorales PCR/ peritoneal fluid<br>PCR and sequencing ITS rDNA/ FFPET/colic sample	*Lichteimia (formerly Absidia)* sp.	*C. glabrata* peritonitis	IV L AMB	Colectomy	Alive
262	Kyuno et al.<br>Ito et al.<br>Same patient	IA: intestinal mesentery, +<br>Abd skin, stoma, wall muscles, spleen, stomach, bladder, kidney	Intestinal mesentery sample<br>Autopsy: stoma, skin, abdominal wall muscles, spleen, stomach, bladder, kidney	Negative culture/intestine, abdominal skin, muscle<br>Identification<br>PCR + sequencing (target gene NS) /FFPET autopsic iliac vein thrombus	*R. arrhizus (formerly oryzae)*	NR	None	Surgical wound debridement,	Dead
263	Meshram et al.	Spleen, stomach	NR	Positive culture/spleen abscess, gastric sample	*Rhizopus* sp.	*Klebsiella* sp. septicaemia	IV L AMB 5mg/kg/d	Splenectomy, gastric ulcer resection	Dead 2 d after surgery
264	Ralaizanaka et al.	Stomach, intestine (rectum)	Gastric Bx	Positive rectal fistula secretions culture	*Rhizopus* sp.	NR	IV L AMB 5mg/kg/d, 6 w	Temporary colostomy	NR, alive at 6 w
265	Banerjee et al.	Intestine, mesenteron	NR	Positive DE Intestinal + mesenteric tissue	NI	NR	IV L AMB, total dose 200 mg	Terminal ileum, cecum, ascending colon, half of transverse colon resection	Dead
266	Rathi et al.	Stomach, intestine	Gastric, intestinal samples	None	NI	NR	None (post mortem diagnosis)	Gastric necrosectomy,<br>> 7 d of admission	Dead, 3 d after surgery<br>Post-mortem diagnosis
267	Li et al.	Adrenal gland then (5M) liver	Adrenal gland, liver samples	Negative culture/Intra-operative bile	NI	NR	IV L AMB, 3M, total dose 3 g then ITZ 100 mg/d, 4M	Partial hepatectomy	Alive
268	Rabin et al.	Disseminated: hearth, lungs,thyroid, kidneys, stomach	Autopsy	None	NI	NR	None	None	Dead
269	Straatsma et al.	Disseminated: hearth, lung, thyroid, liver, spleen, Gl, kidneys	Autopsy	None	NI	NR	None	None	Dead

270	Straatsma et al.	Disseminated: hearth, lung, kidney, spleen	Autopsy	None	NI	NR	None	None	Dead
271	Straatsma et al.	Disseminated: hearth, lungs, GI, liver, spleen, kidneys	Autopsy	None	NI	NR	None	None	Dead
272	Agger et al.	Disseminated: lung, gastrointestinal	Autopsy: lung, GI mucosa	Positive culture/pulmonary tissue	*Mucor sp.*	C. albicans peritonitis	IV AMB 25 mg	None	Dead, post-mortem diagnosis
273	Virmani et al.	Disseminated: hearth, lung, liver, kidneys, spleen, adrenal, brain	Autopsy	None	NI	NR	None	None	Dead
274	Virmani et al.	Disseminated: hearth, lung, kidneys, GI, skin	Autopsy	None	NI	NR	None	None	Dead
275	Gupta et al.	Disseminated: intestine, mesentery, peritoneum, spleen, kidneys, lung	Autopsy	None	NI	NR	None	None	Dead
276	Nakamura et al.	Disseminated: peritoneal, spleen, brain, lung, heart	Autopsy: lungs, heart, liver and spleen	Post mortem cultures: peritoneal, brain, blood	*R. microsporus*	NR	IV AMB, 12h before dead	None	Dead, post-mortem diagnosis
277	Nagy-Agren et al.	Disseminated: liver, spleen, kidney, thyroid, bone marrow	Autopsy	None	NI	HIV Oropharyngeal thrush	None (post mortem diagnosis)	None	Dead, post-mortem diagnosis
278	Singh et al.	Disseminated: oesophagus, stomach, lung, hearth, aorta	Oesophageal, gastric samples Autopsy: lung, hearth, aorta	Positive culture/oesophageal, gastric samples	*Rhizopus* sp.	NR	IV L AMB 6 mg/kg/d	Total esophagectomy, hemigastrectomy	Dead, 2 w after re-transplantation
279	Stoebner et al.	Disseminated: Rhinofacial, liver, kidney	Autopsy: kidney, liver	Positive skin sample culture	Rhizopus sp.	NR	IV AMB 1 mg/kg/d	None	Dead

280	Tsaousis et al.	Disseminated: liver, brain (probable)	NR	Positive DE+Culture / liver aspirate	*Rhizopus* sp.	NR	IV L AMB 1,5 mg/kg/d, 40 d then ITZ 100 mg x 3/d, 3 M	None	Alive
281	Sehgal et al.	Disseminated: rhinocerebral (1st site), intestine (colon), peritoneal	Rhinocerebral samplesthen (2 w) peritoneal fluid, colon sample	None	NI	NR	IV AMB 1 mg/kg/day	Abdominal surgical debridement	Dead
282	Gimeno-Garcia et al.	Disseminated: stomach, pancreas, lung	Autopsy: stomach, pancreas, lung	None	NI	*P. aeruginosa* bacteraemia	None (post-mortem diagnosis)	None	Dead, post-mortem diagnosis
283	Alexander et al.	Disseminated: intestines, spleen, liver, kidney, lung, heart	1, kydney sample Autopsy (Kidney, spleen, liver, intestines, lung, heart)	Positive culture /Retroperitoneal pocket, abdominal tissues ITS2, ITS2 28S rDNA regions PCR and sequencing	*A. elegans*	NR	IV LC AMB + MCF	Transplant nephrectomy, multiple debridement	Dead
284	Arena et al.	Disseminated: intestine (stomach, jejunum), kidneys, lung	Autopsy	None	NI	NR	None	None	Dead, post-mortem diagnosis
285	Gurevich et al.	Disseminated: liver, pericardial fluid	Liver Bx then liver explant sample	Negative cultures/liver abscess and pericardial fluid<br>DG and identification: ITS rDNA regions PCR + sequencing/punction samples	*R. arrhizus (formerly oryzae)*	Ascites infection (coagulase negative *Staphylococcus* and *C. krusei*)	IV L AMB + PSZ<br>then addition of CSP<br>then PSZ 12 M	Re-transplantation (w13 after	Alive
286	Nam et al.	Disseminated: lung, peritoneal, spleen, brain, heart	Lung Bx,<br>peri- allograft, splenic abscess drainage fluid	None	NI	NR	IV L AMB 5 mg/kg/d, 33 d then 10 mg/kg/d, 2d	Peri- allograft, splenic abscess drainage 33d under AFT	Dissemination + peri- allograft, splenic abscess increasing under AFT<br>Dead
287	Alqhamdi et al.	Disseminated: lung, spleen	Lung Bx and sample, spleen sample	Positive culture/ lung Bx and sample, spleen sample	*Mucorales*	NR	IV AMB +MCF, 2 w	Lobectomy, splenectomy, stomach wedge resection	Alive
288	Prohaska et al.	Disseminated: stomach, intestine (colon), pancreas, kidney, lung, myocardium	Autopsy	Negative culture of BAL	NI	BAL: *C. glabrata*, *P. jirovecii*, CMV, *L. pneumophila*<br>Disseminated aspergillosis	None (post-mortem diagnosis)	None	Dead, post-mortem diagnosis

289	Evert et al.	Disseminated: heart, intestine	Autopsy: heart, intestine	Identification: PCR and sequencing ITS rDNA/ fresh-frozen tissue (FFT) or FFPET intestine and myocardium	*Rhizopus microsporus /azygosporus*	NR	None	None	Dead, septic MOF, post-mortem diagnosis
290	Sami et al.	Disseminated: Bone marrow, ascites	NR	Positive DE + negative culture/Bone marrow Positive culture / Ascites fluid	*Mucor* sp.	Pseudomonas ascites infection	PO PSZ	None	Dead, 5 d after AFT start

Table S2. Table S2. Summary of diagnostic, treatment and outcome reported in the 290 cases of gastrointestinal (GI) and intra-abdominal (IA) mucormycosis that were included in the study.

Rvw, reviewed by. Bx, biopsy. FFT, fresh-frozen tissue, FFPET, formalin-fixed paraffin-embedded tissue. Abs, antibodies. DG, diagnosis. DE, direct examination. NR, not reported. NI, not identified. NS, not specified. TB, tuberculosis. AFT, anti-fungal treatment. d, day. w, week. M, month. IV, intravenous. PO, *per os*. AMB, amphotericine B. L AMB, liposomal amphotericine B. LC AMB, lipid complex amphotericine B. PSZ, pozaconazole. ITZ, itraconazole. IZV, izavuconazole. KTZ, ketoconazole. FCZ, fluconazole. MCF, micafungine. CSF, caspofungine. POT, post-organ transplant. MOF, multi-organ failure.

a. Outbreak/wooden tongue depressors
b. Additional personal data

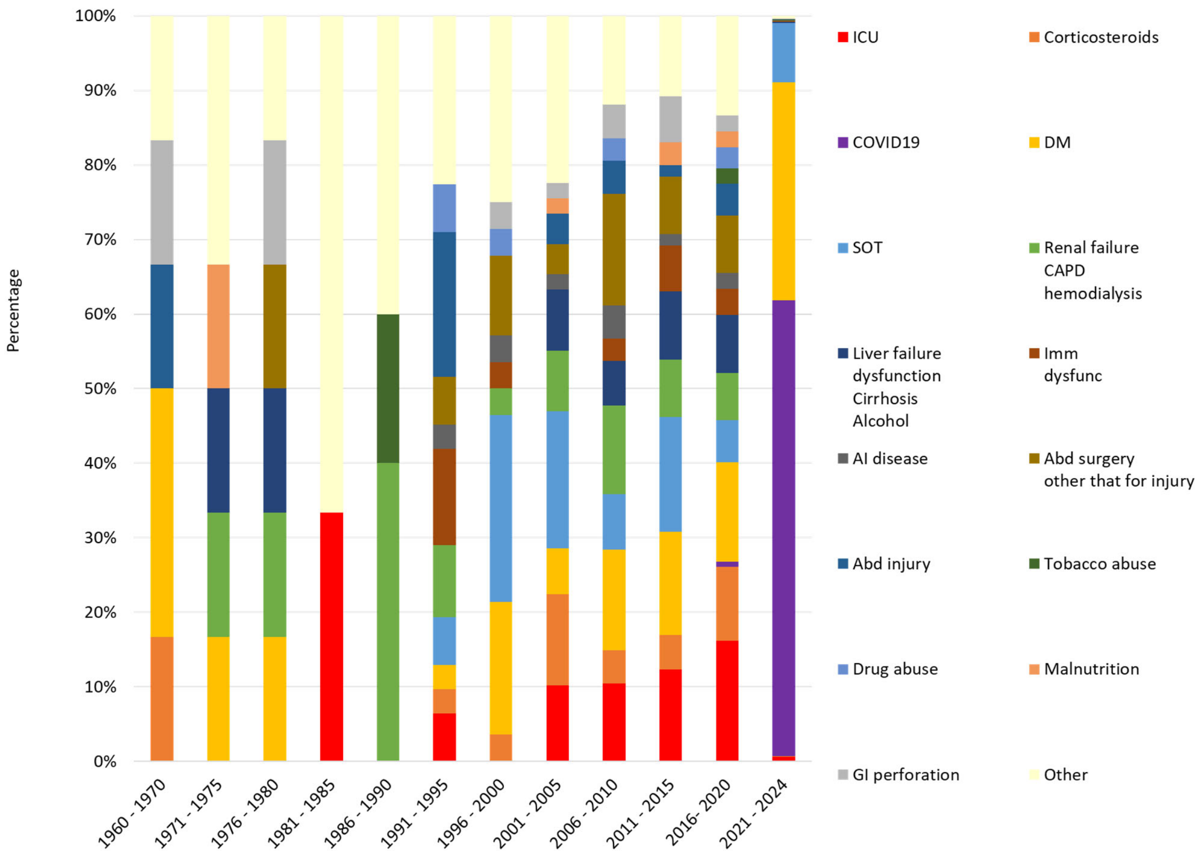

Figure S1. Distribution (%) of the underling diseased and conditions according to the decade of publication. "Other" category includes:

- GI/IA, pelvic condition (n = 25): gastritis (n = 3), gastric erosion, adenocarcinoma, portal gastropathy, gastroparesis, peptic ulcer history, gastro-enteritis, *Salmonella* enteritis, *C. difficile* colitis, pan-colitis, digestive adenocarcinoma, diarrhoea, pancreatitis, peritoneal infection/peritonitis history (n = 6), breach in CAPD sterile technique, percutaneous drainage of renal collection, postpartum haemorrhage, testicular cancer + chemotherapy, sider bite (n = 1).

- Thoracic/pulmonary condition (n = 18): ARDS (9), COPD (n = 3), respiratory failure, emphysema, H1N1 pneumonia, pulmonary tuberculosis, pleural infection, chest stab, thoracic surgery (n = 1)

-Infection history (n = 4): typhoid fever (2), rhino-facial mucormycosis, *P. falciparum* malaria (n=1)

- Iron overload / transfusions (n = 5)

- Miscellaneous (n = 12): tobacco use (n = 5), burn (n = 2), back trauma, pesticide ingestion, cytopenia, haemolytic uremic syndrome, motor hemiplegia (n = 1). None (n=5).

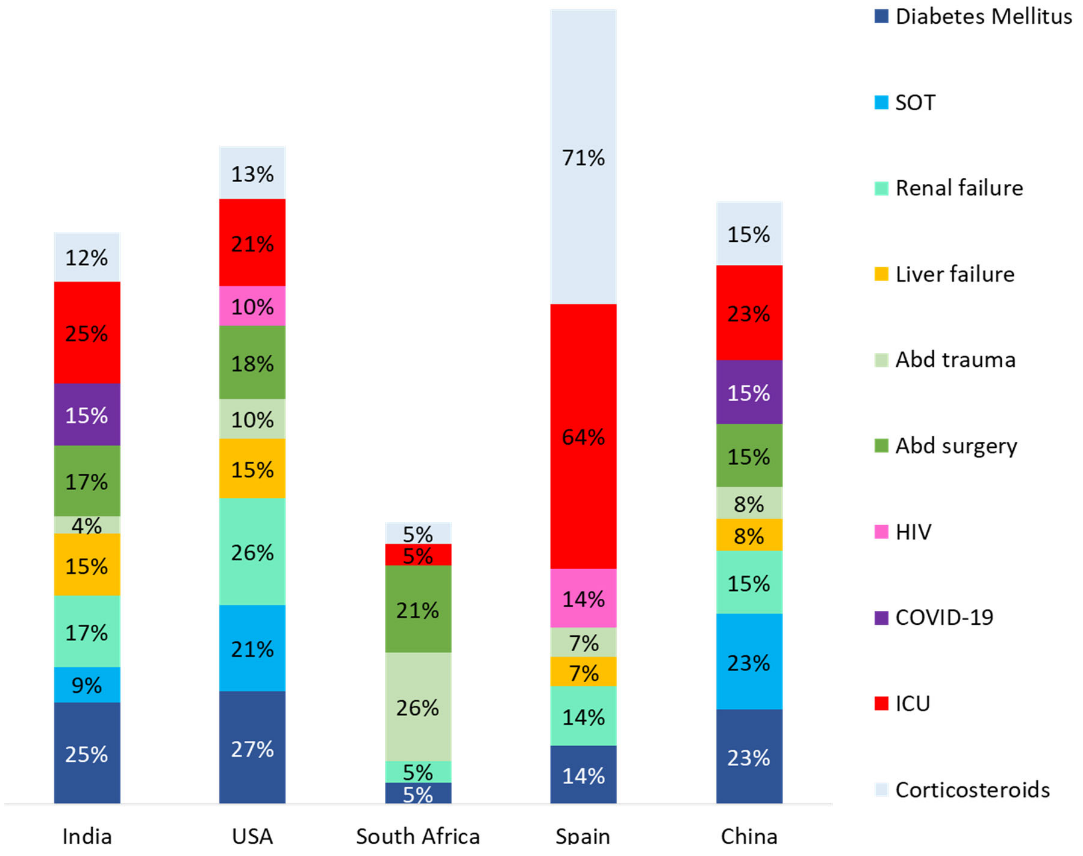

Figure S2. Distribution (%) of main underlying diseases and conditions according to the country for the five countries with more than 10 reported cases.
